# Development and validation of RdRp Screen, a crystallization screen for viral RNA-dependent RNA polymerases

**DOI:** 10.1242/bio.037663

**Published:** 2019-01-15

**Authors:** Federica Riccio, Sandeep K. Talapatra, Sally Oxenford, Richard Angell, Michela Mazzon, Frank Kozielski

**Affiliations:** 1 Department of Pharmaceutical and Biological Chemistry, UCL School of Pharmacy, 29-39 Brunswick Square, London, WC1N 1AX, United Kingdom; 2 Translational Research Office, UCL School of Pharmacy, 29-39 Brunswick Square, London, WC1N 1AX, United Kingdom; 3 UCL MRC Laboratory for Molecular Cell Biology, Gower Street, London, WC1E 6BT, United Kingdom

**Keywords:** Dengue virus, Crystallization screen, RNA-dependent RNA polymerase, Flavivirus, Antiviral drug discovery

## Abstract

Members of the *Flaviviridae* family constitute a severe risk to human health. Whilst effective drugs have been developed against the *hepacivirus* HCV, no antiviral therapy is currently available for any other viruses, including the *flaviviruses* dengue (DENV), West Nile and Zika viruses. The RNA-dependent RNA polymerase (RdRp) is responsible for viral replication and represents an excellent therapeutic target with no homologue found in mammals. The identification of compounds targeting the RdRp of other flaviviruses is an active area of research. One of the main factors hampering further developments in the field is the difficulty in obtaining high-quality crystal information that could aid a structure-based drug discovery approach. To address this, we have developed a convenient and economical 96-well screening platform. We validated the screen by successfully obtaining crystals of both native DENV serotype 2 and 3 RdRps under several conditions included in the screen. In addition, we have obtained crystal structures of RdRp3 in complex with a previously identified fragment using both soaking and co-crystallization techniques. This work will streamline and accelerate the generation of crystal structures of viral RdRps and provide the community with a valuable tool to aid the development of structure-based antiviral design.

## Abstract (precis)

**Summary:** We have developed a 96-well crystallization screen for viral RNA-dependent RNA polymerases (RdRps). The screen provides a convenient platform for streamlining the crystallisation of RdRps and implementing structure-based drug discovery programs.

## INTRODUCTION

*Flaviviridae* are a family of enveloped, positive single stranded RNA viruses. The genus *Flavivirus*, of the *Flaviviridae* family, counts over 70 different viruses (Fields et al., 2007; Kuno et al., 1998), including Dengue virus (DENV), Japanese encephalitis virus (JEV), tick-borne encephalitis virus (TBEV), West Nile virus (WNV), yellow fever virus (YFV) and Zika virus (ZIKV). Most of these viruses are arthropod-borne and can cause widespread morbidity and mortality. For instance, infection with DENV, which is estimated to affect 390 million people annually (Bhatt et al., 2013), can lead to an ample range of clinical manifestations, from mild fever to fatal dengue shock syndrome (Rajapakse, 2011), while infection with ZIKV has recently been shown to be responsible for the sudden surge in the number of cases of microcephaly and neurological abnormalities in new-borns, and for several cases of Guillain-Barré syndrome (Dyer, 2015; Oliveira Melo et al., 2016). No antivirals are currently available and vaccines are limited to YFV, JEV and TBEV. The vaccine currently licensed for DENV (Dengvaxia, Senofi-Pasteur) only has limited efficacy against some DENV serotypes, and concerns have been raised over its administration to children and seronegative individuals (Aguiar et al., 2016). In the absence of safe and effective vaccines, and given the risk of emergence of new flaviviruses, as demonstrated by the recent re-emergence of ZIKV, the development of antivirals against this group of viruses becomes ever more important.

The flavivirus genome of ∼11 kb is translated into a single polyprotein which is processed into three structural (envelope, membrane and capsid) and seven non-structural proteins (NS1, NS2A, NS2B, NS3, NS4A, NS4B, NS5). NS5 is the largest and most conserved protein, with members of the flavivirus genus sharing approximately 60–65% sequence similarity (Lim et al., 2015).

DENV NS5 (∼900 aa) is comprised of a methyltransferase (MTase) domain (∼250 aa) at the N-terminus, mainly responsible for RNA cap formation during viral replication (Egloff et al., 2002; Ray et al., 2006), and an RNA-dependent RNA polymerase (RdRp) domain at the C-terminus (∼600 aa). The RdRp is mostly known for its role in virus replication (Selisko et al., 2014). It functions by replicating the viral genomic +RNA into uncapped –RNA, leading to the formation of a double-stranded RNA intermediate, and then using the –RNA template to synthesize new +RNA copies of the viral genome (Malet et al., 2008). In addition, the RdRp plays an important role in escaping the host immune response by blocking IFN type I signalling through binding the transcription factor STAT2 and promoting its degradation (Ashour et al., 2009; Mazzon et al., 2009).

**Fig. 1.**
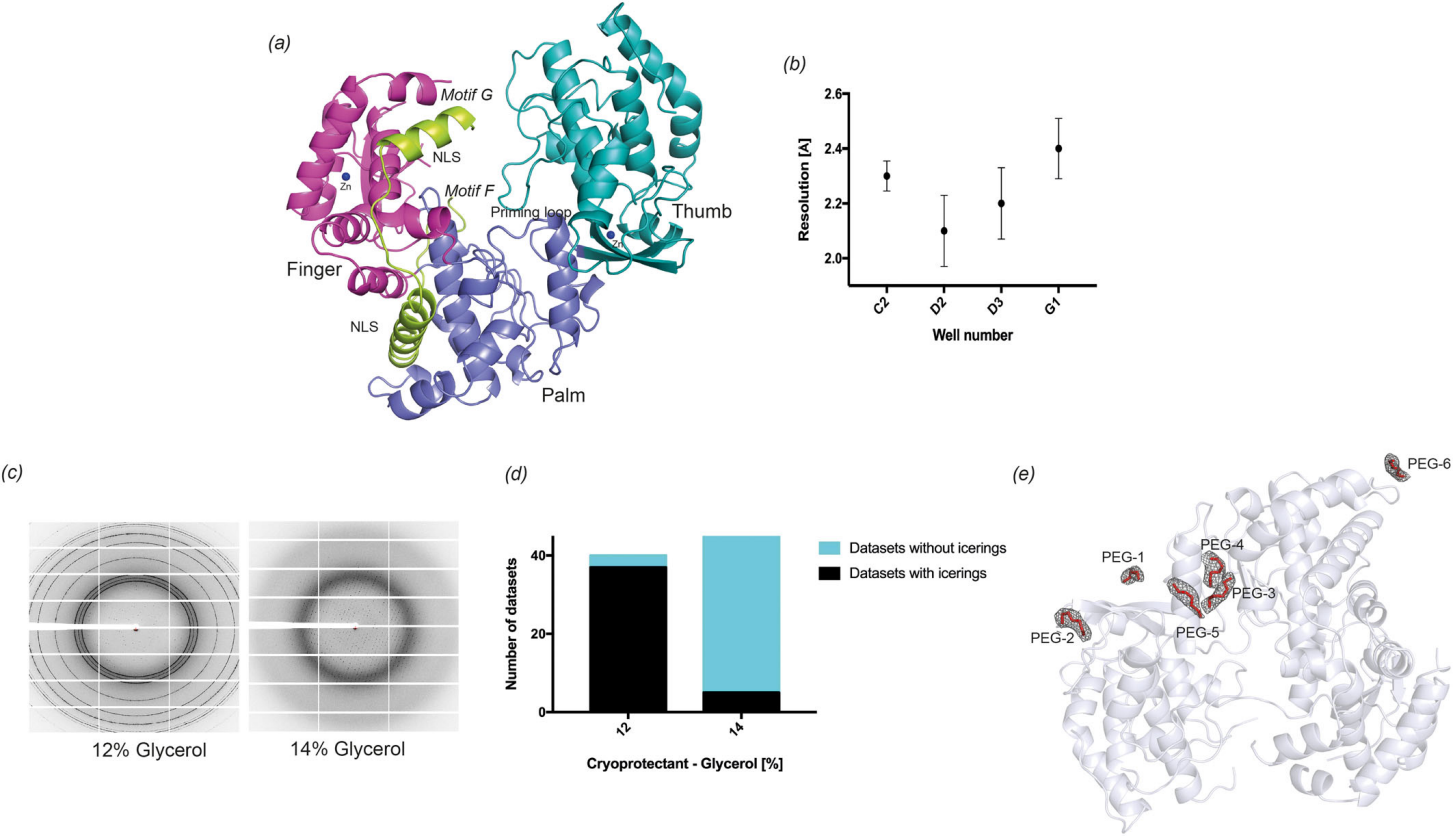
**Representative details of optimized cryo-conditions.** The structure of dengue RdRp and the location of PEG-ions in the structure. (A) The overall structure of the RdRp domain of dengue virus serotype 3. The different secondary elements represent the thumb (turquoise), finger (magenta), palm (purple) and NLS regions (green). The two Zinc atoms are represented as blue spheres. (B) Representative diffraction pattern of RdRp crystals in the presence of either 12% glycerol or 14% glycerol as a cryoprotectant are shown. 12% glycerol shows ice rings correlated with a decrease in resolution, which prompted us to investigate 14% glycerol as a cryoprotectant (*n*=10, mean±s.d.). (C) Bar diagram of quantitative representation of number of datasets obtained with 12 (*n*=39) and 14% (*n*=41) glycerol as cryoprotectant that either had ice rings (black) or no ice rings (blue). (D) The PEG ions coordination and their electron density omit map (coloured in grey) contoured at 1 σ. The numbering of PEG is based on the number of PEG molecules present in different structures. For example, a structure with four PEG ions will contain PEG-1 to PEG-4. Figures B and C were prepared using PyMOL (Schrodinger, 2015). (E) Various PEG molecules are located at the surface of RdRp and mediate interactions with symmetry-related molecules.

The overall structure of the RdRp domain consists of three main subdomains known as the ‘fingers’, ‘palm’ and ‘thumb’ (Fig. 1A). These subdomains are made up of seven conserved motifs (A to G) important for RNA binding and replication (Sousa, 1996; Malet et al., 2007; Yap et al., 2007). Motifs F and G are believed to interact with the RNA template (Iglesias et al., 2011) and with nucleoside triphosphates (NTP) (Sousa, 1996) for RNA elongation. It has been proposed that DENV RdRp undergoes a conformational change from a ‘closed’ initiation complex, bound to single-stranded RNA, to an ‘open’ elongation complex, bound to double-stranded RNA. Not surprisingly, sections of the flexible loops from motifs F (residues 455–468) and G (residues 406–417) are disordered and not observed in the apo-structures (Yap et al., 2007). Structures of dengue RdRp have only been solved in the closed conformation (Noble and Shi, 2012). Interestingly, in the ligand bound structure (PDB ID: 3VWS; Noble et al., 2013) one region involved in ligand binding near motif G has the whole motif present although the overall structure is still in the closed conformation.

Being essential for viral replication and with no equivalent in host cells, DENV RdRp represents an attractive target for drug development. Also, given its structural and conformational conservation among the various serotypes (Rawlinson et al., 2006), the RdRp domain represents one of the most viable targets for the development of direct-acting DENV antivirals. The clinical use of inhibitors against the HBV, HCV and herpes virus polymerase as well as the HIV reverse transcriptase, has validated viral polymerases as therapeutic targets (De Clercq et al., 2006). At present, the only clinically approved antiviral therapy targeting a *Flaviviridae* RdRp is used for the treatment of HCV infections (Bonaventura and Montecucco, 2016; Younossi et al., 2016). The study of antivirals targeting DENV RdRp has led to the identification of a few potential candidates, but further work is needed to develop a viable drug (Noble et al., 2013, 2016; Yokokawa et al., 2016). In order to further advance drug development efforts against the RdRp of DENV and other *Flaviviridae*, determining the structures of the RdRps for rational drug design is of crucial importance.

To date, several RdRp structures of various members of the *Flaviviridae* family have been determined either in the apo state or in complex with inhibitors or fragments (Noble et al., 2013, 2016). Apo structures of RdRp provide information about structural similarities and differences within the family, which has to be taken into consideration during the various phases of the drug discovery process. In contrast, crystal structures of RdRps in complex with small molecules or fragments provide insights into inhibitor binding pockets for the development of new antivirals.

The commercial screens currently available for crystallization trials require extensive screening for crystals, which is time consuming, cumbersome and expensive. No targeted crystallization screen for viral RdRp proteins is currently available. In order to address these limitations, we have developed a fast and cost-effective RdRp screen with the intent of facilitating crystallization of RdRps from different viruses either alone, or in complex with inhibitors or fragments. Our aim was to rationalize the crystallization processes for different RdRps, by searching the PDB and the literature for crystallization conditions of all known RdRp structures, and to develop a screen specifically designed for crystallization of these proteins. We devised a crystallization screen comprising of 96 different conditions, optimized for use in 96-well plate format. We have further verified these screening conditions by crystallizing the RdRps of DENV serotypes 2 and 3. Furthermore, we obtained RdRp3 in complex with the fragment PC-79-SH52 (Noble et al., 2016) using our novel screen under both soaking and co-crystallization conditions.

## RESULTS

### Data mining and analysis of the PDB

**Fig. 2.**
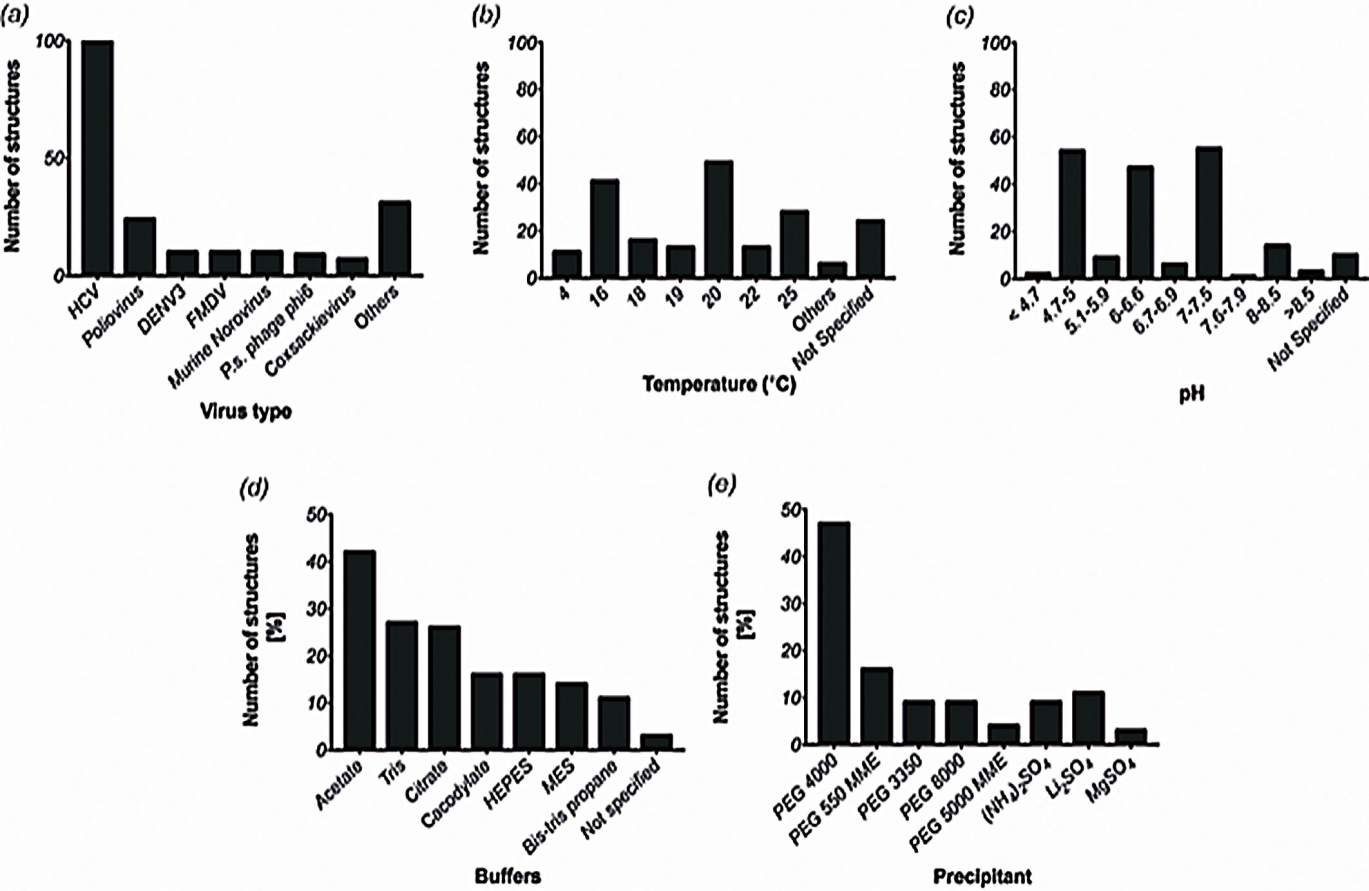
**Analysis of PDB data.** (A) Deposited structures of RdRp domains of different viruses. HCV has the highest number of PDB entries (∼100 structures), whereas other major viruses have about 10–20 PDB entries. (B) A range of temperatures have been employed to obtain crystals for RdRp domains with 20°C being the most common one followed by 16°C and room temperature (25°C). Other temperatures have been used sparingly to obtain crystals. (C) Although a wide range of pH values have been successful in obtaining various crystals, pHs closer to physiological pH values have been more successful than others. Bar diagram representation of percentage structures obtained (D) under various buffers and (E) obtained with various precipitating agents from the PDB data analysis.

Information about each viral RdRp was retrieved. This included the PDB ID, crystallization method, pH, the crystal growth procedure and conditions, the resolution, and space group for each entry. A crystallization dataset with 201 unique entries was created as shown in Table S1. The RdRp domains deposited in the PDB database originate from 19 different viruses. The most studied virus is HCV, counting 49% of all entries, reflecting the importance of HCV RdRp as therapeutic target of new drugs introduced to the market, followed at a significant distance by Poliovirus (12% of the entries). Other extensively studied viruses are DENV serotype 3, foot and mouth disease virus, and murine norovirus, each representing 5% of the entries (Fig. 2A). The success of specific inhibitors against HCV RdRp underpins the importance of this novel screen for structure-based drug design targeting the RdRp of other viruses of significant public health concern.

To generate the data set, analysis of the crystallization conditions was carried out taking into account the precipitant used, the buffer, as well as its pH, the salt composition and the crystallization temperature (Table S1). Unfortunately, in about 15% of entries, the information deposited in the PDB or in the corresponding manuscript did not include exact crystallization conditions.

A range of temperatures from 273K (0°C) to 303K (30°C) were used to crystallize RdRps. The most used temperatures were 293K (20°C; 28%), 289K (16°C; 24%) and 298K (25°C; 16%). Only very few structures were determined at 277K (4°C) (1%) and above 298K (30°C) (0.5%). For 12% of entries there was no specified crystallization temperature (Fig. 2B).

We observed that the majority of crystals were obtained in a range of pH values between 4.6 and 10.0. Most of the structures were determined at a pH between 7.0–7.5 (29%), and between 4.7–5.0 (28%), followed by pH ranges 6.0–6.6 (25%). The most used single pH values were 7.5 (15%), 7.0 (13%), 4.9 (11.5%) and 5.0 (11%). For 6% of the entries there was no specified pH value (Fig. 2C).

The buffers used to maintain this pH principally included acetate buffer (42%), tris (hydroxymethyl)amino-methane (Tris, 27%), citrate (26%), cacodylate (16%), 2-[4-(2-hydroxyethyl) piperazine-1-yl] ethane-sulfonic acid (HEPES, 14%), 2-(N-morpholino) ethanesulfonic acid (MES, 14%), and Bis-tris propane buffer (11%). Around 3% of entries did not specify the buffer used (Fig. 2D).

The precipitating agent most commonly employed for crystallization was polyethylene glycol (PEG), used in 88% of the cases. A variety of PEGs with different molecular weights were used including PEG 4000 (47%), followed by PEG 550 monomethyl ether (MME, 16%), PEG 8000 and PEG 3350 (both 9%), PEG 400 (7%) and PEG 5000 MME (4%). The second most employed precipitating agent were sulfate salts, in around 23% of the entries, including ammonium sulfate in 9% of the cases, lithium sulfate (11%) and magnesium sulfate (3%) (Fig. 2E).

Cations and additives also seemed to be important for the formation of crystals. The most used monovalent cation is Na^+^ (51%) from acetate buffer, sodium chloride and Na/K tartrate, followed at some distance by Li^+^ (6%) (e.g. lithium sulfate and lithium nitrate) and by K^+^ (3%) included in Na/K tartrate, potassium fluoride and potassium phosphate. Divalent cations commonly used are Mg^2+^ (12%) such as magnesium chloride, acetate buffer and magnesium sulfate, and Mn^2+^ (9%) from manganese chloride. Less used is Ca^2+^ (3%) such as in acetate buffer and calcium chloride.

In the case of additives, glycerol was used in 44% of all crystallization conditions, but was also employed as a supplement during protein purifications. This indicates that glycerol was used as an additive to increase protein solubility, to decrease the number of nucleation centres and as a cryo-protectant. Other alcohols such as 2-propanol (6%), ethylene glycol (2%) and 1,6-hexanediol (1%) have been used as additives in the crystallization of various different RdRps.

### Design of the RNA polymerase screen

Based on the most successful conditions identified in the PDB analysis, the RNA polymerase screen was designed to include 96 crystallization conditions covering the widest possible variety of precipitants, buffers and pH values, salts and additives. The identified conditions were initially grouped based on the precipitant used and, within each group, ordered by the precipitant concentration. Five different precipitants were selected for the screen. Sparingly used precipitants such as sodium potassium tartrate, ammonium sulfate, etc. were employed to populate the majority of rows A and B of the 96-well screen format. According to the PDB analysis, different concentrations (1–40%) of PEG chains of varying lengths (400–20,000) were most abundantly used to obtain RdRp structures from distinct viruses. Therefore, about 70 of the 96 formulated conditions contain some form of PEG as a precipitant.

Next, we decided to include in the screen a pH distribution between pH 4.7 and pH 10.0, in order to cover the pH range most frequently used in the PDB dataset as extensively as possible. For each individual precipitating agent, we moved from the lowest pH to the highest pH. This trend has been maintained in a serpentine manner with the majority of low pH values towards the lower denomination column and the higher pH values in the higher denomination columns. Few exceptions to this rule are due to space constraints, as we chose to give the screen a larger variation in the use of precipitants rather than the pH. As final criteria, the salt used in each formulation was considered in order to further expand the conditions for the screen. Based on our PDB analysis, the most abundant monovalent and divalent cations that we included in our formulation are Na^+^ and Mn^2+^, respectively. However, we also tried to include the largest number of salts and concentrations possible for each precipitant of the screen. Additives have also been included in some conditions in minute amounts and we placed the same condition with and without additives in adjacent wells. The complete formulation of the RdRp screen is shown in Table 1. The details of well compositions with volumes of each component used are shown in Table S2. The source and stock solutions of each chemical used in the screen are listed in Table S3.

**Table 1.**
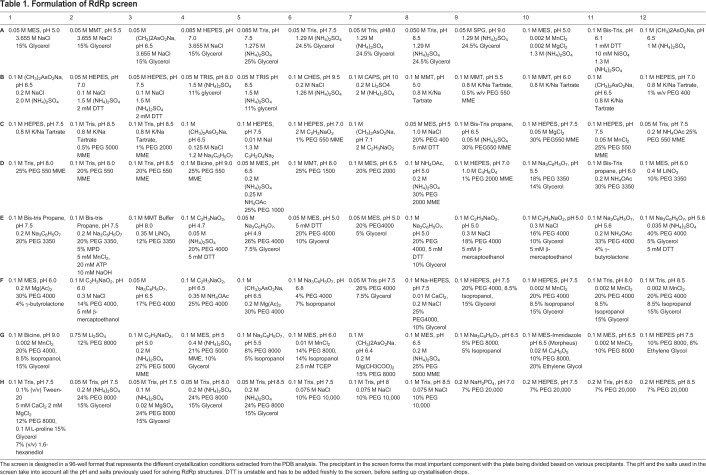
**Formulation of RdRp screen**

### Crystallization results

**Fig. 3.**
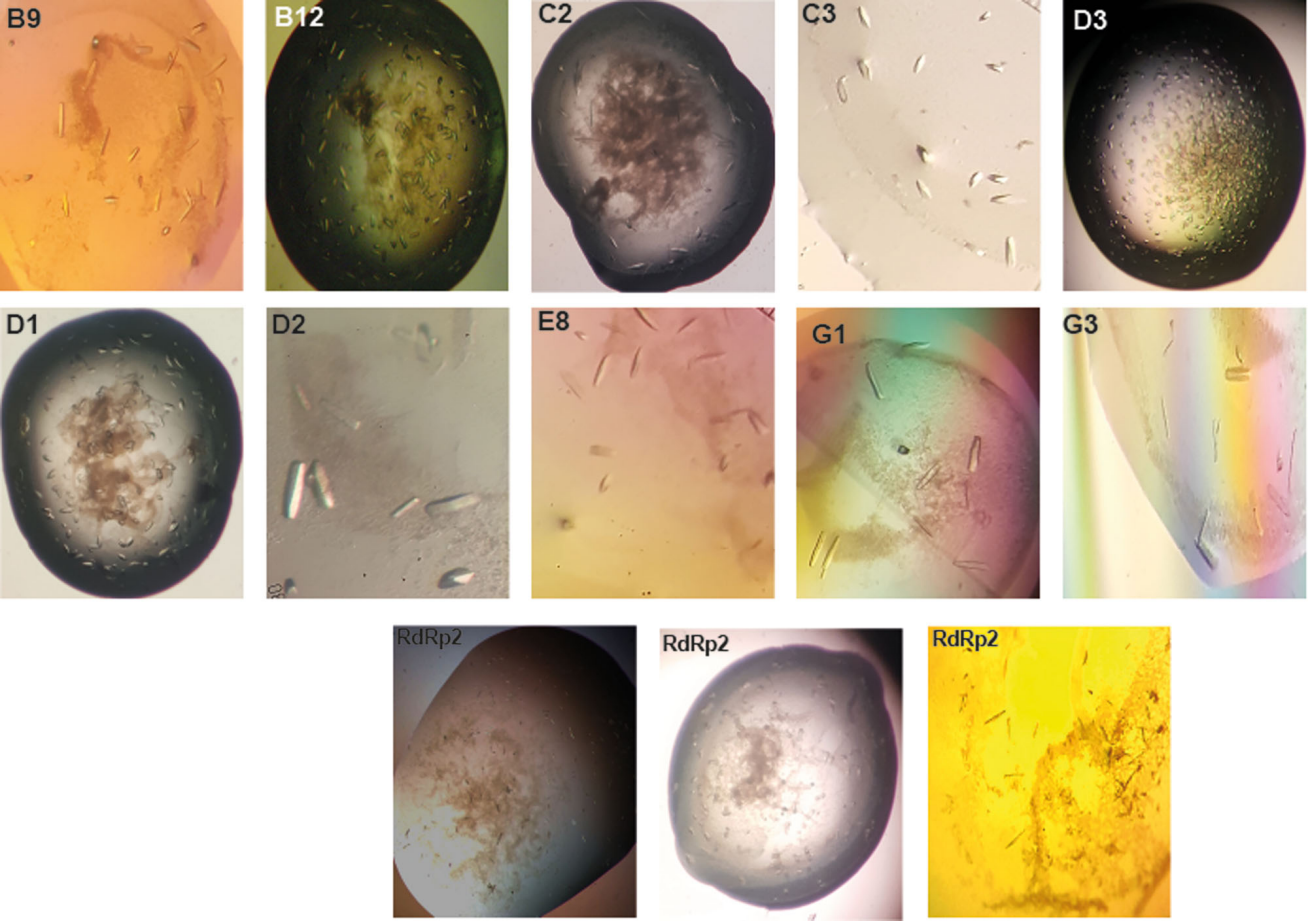
**Light micrographs of some of the crystals obtained with the RdRp screen.** The images shown represent a selection of crystals obtained directly from the screen, without further optimization of the conditions. The well numbers for each crystal image are displayed at the top left corner. The last panel represents RdRp2 crystals obtained in wells F10, F11 and F12, respectively. Magnifications depicted here may differ among the crystal images. Crystal sizes typically vary from 20–300 μm.

To validate the RdRp screen developed, we tested whether we could obtain crystals of DENV serotype 3 RdRp. This protein appeared to be a highly promiscuous crystallizer: crystals were obtained in 36 distinct conditions, more than a third of all crystallization conditions provided in our screen. A selection of photos of the crystals is shown in Fig. 3. Most of the crystals grew within 2–4 days to a size sufficient to examine their diffraction potential; however, in order to obtain larger crystals, crystals obtained from the initial screen in nano-drops were next grown under the same conditions in micro-drops, without further optimization.

The most successful precipitant agent was found to be PEG (in accordance with the PDB analysis), present at an average concentration of 20% in 24 of the 36 conditions that yielded crystals. Specifically, PEG with a chain length of 4000 was most prevalent amongst the successful conditions. The pH range covered by conditions containing PEG varied largely between 4.7 and 8.5, but the majority of the crystals were obtained at pH values of 5.0, 6.5 and 8.0. There appears to be no correlation between the formation of crystals and the buffer used to maintain the pH, with a variety of buffers used in the successful conditions, including Tris, Bis-Tris propane, MES, acetate and citrate buffers. Similarly, salt in the crystallization conditions seemed to have a minimal effect on crystal formation.

Crystals obtained in PEG-containing conditions were observed to be variable in shape and size, with most appearing as single crystals. Thirteen conditions out of the 24 provided high quality data better or equal to 3.0 Å to determine their structures, whereas the remaining crystals either showed no diffraction or diffraction at a much lower resolution not suitable for structure solution (Table S4).

The second most successful precipitant agents for obtaining protein crystals were salts such as, for example, sodium-potassium tartrate, sodium chloride and sodium malonate. All but one yielded resolution of less than 3.0 Å. Four conditions containing ammonium sulfate generated crystals, from one of these we were able to collect diffraction data and solve the structure at 3.0 Å. Others conditions had crystals which were either too small to measure at an X-ray source, or diffracted to a lower resolution needing further optimization. The pH range covered by these conditions was between 6.0–9.5 and was maintained using a variety of buffers [Tris, HEPES, MMT (DL-malic acid, MES and Tris base in the molar ratios 1:2:2) and sodium cacodylate]. Again, crystals obtained under these conditions were single and variable in shape and size. Diffraction details of the crystals obtained are shown in Table S4. In total, we identified 13 different conditions yielding high quality RdRp crystals, which needed no further optimization to obtain the structures. Protein crystals that would have required further optimization of their crystallization conditions were not pursued further.

### Diffraction data quality analysis from various screen conditions

A summary of the details of crystals obtained is shown in Fig. 3 and Table S4. Previously published crystallization conditions of dengue RdRp3 are represented in the screen in wells C2 (Ref.: PDB ID 2J7U) and D1 (Ref.: PDB ID 4HHJ), both of which resulted in good diffracting crystals with resolution of 2.3 Å and 2.1 Å, respectively. Interestingly, some of the conditions consistently produced crystals within a high-resolution range. The resolution obtained for these screen conditions are summarized in Fig. 1B.

From the diffraction pattern of the 88 crystals measured at either 12% or 14% glycerol we could conclude that 14% glycerol was optimal for cryoprotection of these crystals. 12% glycerol as a cryoprotectant was not sufficient as it resulted in ice rings (Fig. 1C) for the majority of crystals and also resulted in loss of resolution. We have collected complete datasets using both 12% and 14% glycerol and the quantitative representation of the formation of ice rings at different glycerol concentrations is presented in Fig. 1D.

### Data processing and structure determination

Data were processed and reduced using either iMosflm (Battye et al., 2011) or XDS (Kabsch, 2010) and SCALA from the CCP4 suite of programs (Winn et al., 2011). The structures of dengue RdRp3 were solved by molecular replacement (PHASER MR in CCP4 suite) using the native RdRp3 structure (PDB code 4HHJ, Noble et al., 2013) as a search model. All structures were initially refined with REFMAC5 (Murshudov et al., 1997). Electron density and difference density maps, all σA-weighted, were inspected, and the models were improved using Coot (Emsley and Cowtan, 2004). The refinement of the structures was performed using PHENIX (Adams et al., 2010). The calculation of R_free_ used 5% of data. Crystallographic and refinement statistics are given in Table S5. A list of residues missing in the models and the number of PEG ions as well as water molecules identified are summarized in Table S6.

Surprisingly, all crystals except one crystallize in space group C222_1_, indicating that the varying crystallization conditions exert no influence on the crystal packing. The architecture of the RdRp structures obtained from the screen adopts the right-hand conformation consisting of fingers, palm and thumb domain (Yap et al., 2007) (Fig. 1A). The structures attain the same closed conformation with loops from motif G (∼405–420) and motif F (∼450–470) missing in all the structures originating from the screen. All our crystal structures contain two zinc binding pockets represented as blue spheres in the finger and thumb subdomains, respectively (Fig. 1A), which have a tetrahedral coordination geometry as previously described (Yap et al., 2007; Noble et al., 2013).

One structure (PDB ID 2J7U) has one PEG molecule adjacent to Trp823, whereas the second structure (PBD ID 4HHJ) has three PEG entities and one additional P6G (longer ethylene glycol chain) bound to the structure. Twelve of our 13 structures contain PEG molecules but the number of PEGs varies depending on the crystallization condition. We could not establish a relationship between the number of PEG ions present in the structure and the type of PEG used in the crystallization condition. We also could not correlate the number of PEG ions and the resolution of the crystals (Table S5). The role of PEG in the structures is difficult to decipher but the best assumption is that they stabilize the interaction with symmetry-related molecules as most of them are present at the interface of the unit cell and a symmetry related molecule (Fig. 1E).

Overall we conclude that, along with the previous established conditions of dengue RdRp3, we have now found 11 additional conditions, which provide reproducible high-resolution structures without further need for optimization of the crystals. These conditions provide the best diffraction quality crystals with good statistics and therefore can be used for small molecule and fragment crystallization assays. We identified a variety of additional crystallization conditions, but these would require further optimization to obtain structures. Although in this study we obtained a large number of high quality crystals at 18°C, the possibility of using the screen at other temperatures has not been tested for DENV3 RdRp. Until recently there were no crystal structures available for the other serotypes of dengue virus. Recently a structure of dengue RdRp serotype 2 in complex with a small molecule has been published (Lim et al., 2016). In line with the previously published structure we also obtained crystals for dengue RdRp serotype 2 in various conditions at 4°C; however, these crystals were not single (Fig. 3) and diffracted between 4.0 to 5.0 Å resolution (not shown). Optimization of these conditions is now in progress to obtain single high-quality crystals. Therefore, our RdRp-specific crystallization screen can provide an additional avenue and starting point for the discovery of successful conditions for RdRp proteins or RdRp-ligand complexes.

### Description of the structure of the RdRp3-PC-79-SH52 complex obtained by soaking and co-crystallization techniques

**Fig. 4.**
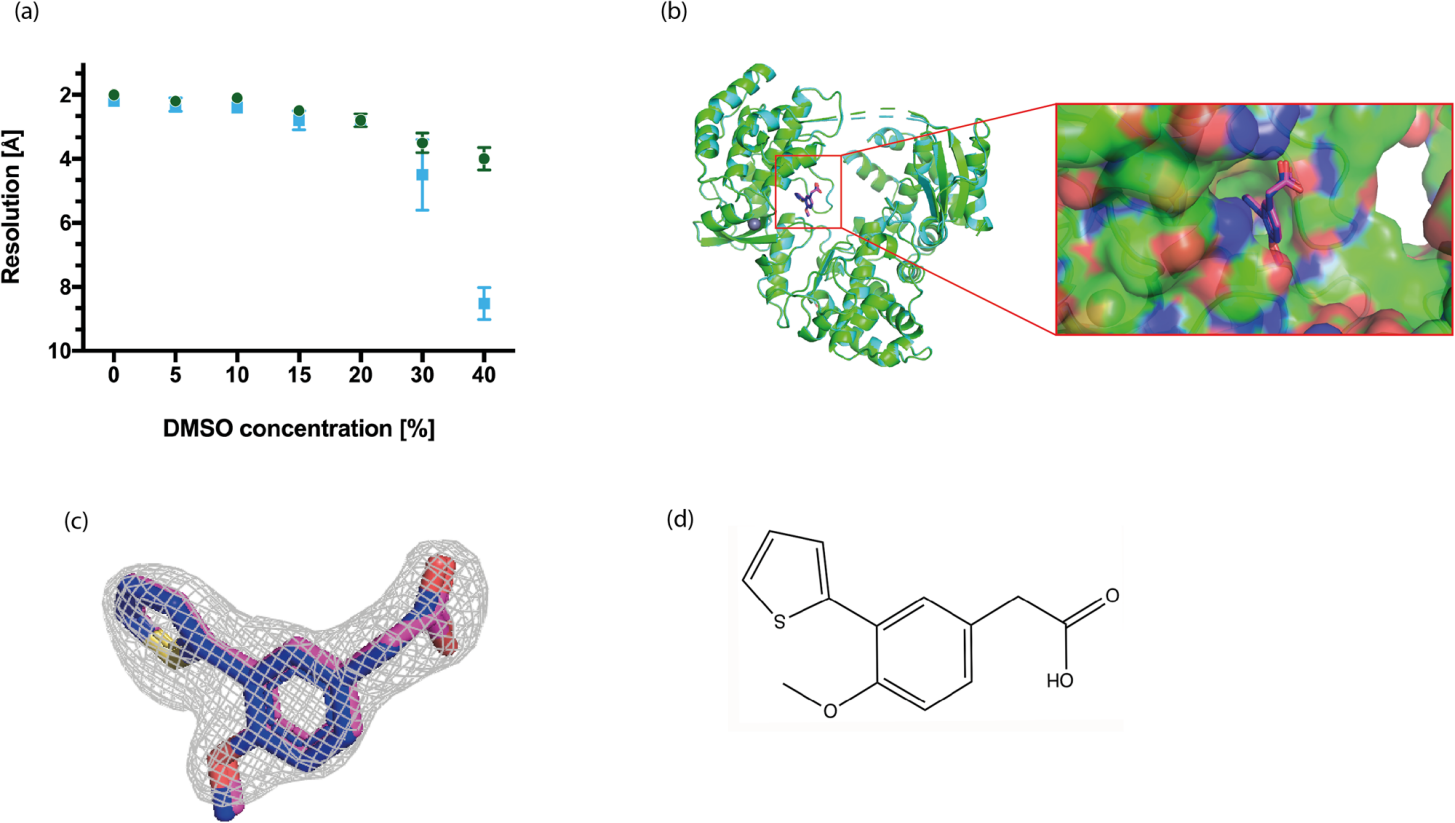
**Structure of RdRp3 in complex with its inhibitor.** (A) Graph representing the DMSO concentration plotted against the resolution using native DENV RdRp3 crystals at 1 h (●) and 3 h (▪) incubation time (*n*=12, mean±s.d.). This allowed us to determine the optimal percentage of DMSO and fragment that can be employed for soaking, data collection and subsequent structure determination. (B) Overall superimposition of the structure of the RdRp3 domain in complex with PC-79-SH52 obtained via co-crystallization (green) and soaking (cyan). There are no obvious differences in the two structures or binding conformations of the inhibitor. Magnification of the inhibitor-binding pocket in the Palm domain as a surface with bound PC-79-SH52. (C) The electron-density of the ligand F_o_-F_c_ difference electron density (contoured at 3σ) is shown as a grey mesh with the inhibitor via co-crystallization (blue) and soaking (magenta). (D) Chemical structure of PC-79-SH52.

The complex structures obtained by co-crystallization (PDB ID: 6H80) and by soaking (PDB ID: 6H9R) are practically identical. They were both obtained using the well condition C1 and C2 from our screen. Both RdRp3 structures lack residues 312–318, 343–354, 406–418, 454–469 and 581–586. The PC-79-SH52 fragment is bound in the palm domain of RdRp protein as previously described (Noble et al., 2016). A significant proportion of the binding is driven by hydrophobic interactions mediated by the thiophene and phenyl ring systems. The sulphur atom of PC-79-SH52 interacts with the side chains of Ala799, Ser796 and Leu511. The thiophene ring points towards the predominantly hydrophobic portion of the inhibitor-binding pocket formed by His711, Met761, Met765, His798 and Trp803. The phenyl moiety also has hydrophobic interactions with Arg729 and Cys709. The carboxyl group forms key hydrogen bonding interactions mediated by water molecules with main chains atoms of Thr794 and Trp795 (Fig. 4; Fig. S1). Overall, the structure of the RdRp3 in complex with the PC-79- SH52 fragment is similar to the previously published structure (PDB ID: 5F3Z; Noble et al., 2016). Therefore we conclude that our screen will also be suitable for future studies on structure-based drug design targeting RdRps.

## DISCUSSION

Certain members of the *Flaviviridae* family are important global pathogens raising significant public health concerns. Their RNA-dependent RNA polymerases represent key targets to treat infections and are intensively studied since there are no mammalian homologues.

The RdRp screen was developed and validated by crystalizing dengue RdRp serotypes 2 and 3, and serotype 3 in complex with a known fragment. PEG molecules of various molecular weights appeared to be the most successful precipitant, in particular PEG 4000. RdRp3 is a promiscuous crystallizer and crystals were obtained in 36 out of 96 distinct conditions, 13 of which did not need any further optimization, yielding crystals diffracting to a high resolution (2.0–3.0 Å). Thirteen complete data sets were collected and structures were obtained from all these data sets.

We believe this study provides a promising platform to screen and crystallize polymerases from other viruses, including emerging RNA viruses such as ZIKV. Studies using the RdRp screen to crystallize the serotypes 1 and 4 of dengue RdRp are underway. Indeed, it will be interesting to observe if optimal crystallization conditions are shared amongst the different serotypes, given their high structural similarity. This screen, which is convenient, fast and cheap, can be used as a first attempt to crystallize novel RdRps to facilitate an understanding of the fundamental processes underlying the replication of viral genomes.

**Table 2.**
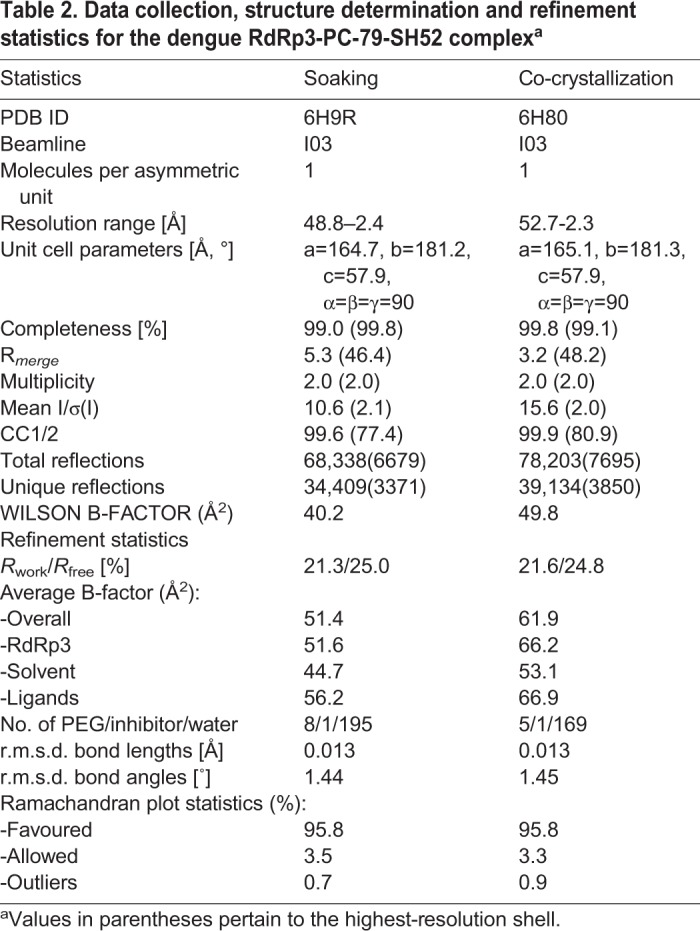
**Data collection, structure determination and refinement statistics for the dengue RdRp3-PC-79-SH52 complex^a^**

Importantly, the screen presented in this study can be used for the crystallization of RdRp-ligand complexes and therefore will support structure-based design to develop novel RdRp inhibitors. The crystal structures of RdRp3 in complex with PC-79-SH52 prove how this new crystallization screen can be used for structure-based drug design against RdRps targets. We have obtained nearly identical complex structures using both co-crystallisation of RdRp3 with PC-79-SH52 or soaking native RdRp3 at high concentrations of the fragment. Therefore, our screen is versatile and flexible in using either of the methods for determining future ligand complex structures of RdRps for structure-based drug design. Traditionally, glycerol has been used as a cryo-protectant for diffraction measurements of RdRp crystals. In our case, we optimized the minimum percentage glycerol required for measuring dengue RdRp3 crystals obtained under various conditions in our 96-well screen. The majority of ligands that are used for soaking or co-crystallization experiments are usually dissolved in DMSO. Interestingly, in our soaking experiments we could show that diffraction measurements of RdRp complexes do not require any additional cryo-protectant. Optimization of the tolerance levels of RdRp crystals at increasing DMSO concentrations resulted in 10% DMSO at an incubation time of 1–3 h for optimal experimental conditions (Fig. 4A). This serves two purposes, first the best condition can be used to soak native crystals with high concentrations of inhibitors/fragments in DMSO, without destroying the crystals and thereby increasing the changes of obtaining co-crystal structures for weak binders. Secondly, the soaking, freezing, and data collection pipeline of RdRp crystals is straightforward.

In summary, our screen provided a variety of novel crystallization conditions leading to highly reproducible and high quality RdRp crystals, which are suitable not only for structure-based design, but also for direct crystallization-based fragment screening. In addition, although we did not obtain high quality crystals for RdRp2, optimising the initial crystallisation condition may lead to suitable crystals for structure determination.

## MATERIALS AND METHODS

### Data mining, analysis of the PDB and the design of the RdRp Screen

The deposited structures of RdRps (alone or in complex with ligands) solved by X-ray crystallography were retrieved and analyzed from the PDB crystallographic database (www.rcsb.org). The details of this database search, which is the basis of this study, are shown in Table S1. The details of the design and development of the screen in 96-well format taking into account all published conditions are shown in Table 1.

### Chemistry

The RdRp inhibitor PC-79-SH52 was synthesized as previously described (Noble et al., 2016; Yokokawa et al., 2016).

### Cloning, expression and purification of Rdrp3 from dengue virus

DENV RdRp serotype 3 (residues 265–900) was amplified from DENV strain D3/SG/05K/2005, previously subcloned into pcDNA3.1 (kind gift from A. Davidson, University of Bristol) using CloneAmp HiFi PCR Premix (Clontech Laboratories, Inc.) as per the manufacturer's instructions (forward primer: AAGTTCTGTTTCAGGGCCCG.AATGCGGAACCAGAAACACCC; reverse primer: ATGGTCTAGAAAGCTTTA.CCAAATGGCTCCCTCCGACTC). DENV RdRp serotype 2 (residues 266–900) was amplified from DENV strain D2/NGC, previously subcloned into pcDNA3.1 (kind gift from A. Davidson, University of Bristol) using the same procedure as for RdRp serotype 3 (forward primer: AAGTTCTGTTTCAGGGCCCG.GGAATTGAAAGTGAGATACCA; reverse primer: ATGGTCTAGAAAGCTTTA.CCACAGGACTCCTGCCTCTTC). After purification of the PCR products, the amplified fragments were cloned by recombination into a pOPINF vector, linearized with *KpnI* and *HindIII* restriction enzymes (New England Biolabs), using the In-Fusion^®^ HD Cloning Kit (Clontech Laboratories, Inc.) as per the manufacturer's instructions. DNA sequencing using a T7 primer was used to verify the presence and correct insertion of the constructs.

*Escherichia coli* BL21(DE3) pLysS cells were transformed with the recombinant plasmid carrying the gene encoding DENV RdRp serotype 3 whereas BL21(DE3) cells were transformed with DENV RdRp serotype 2. Both the proteins were expressed by growing the cells at 37°C in Terrific Broth medium containing 100 mg/l ampicillin until the A_600_ was 0.9–1.2. Protein expression was induced for 22–24 h at 20°C by adding 0.5 mM IPTG (isopropyl-β-D-thiogalactopyranoside). Cells were then harvested by centrifugation at 8000 rpm for 10 min at 4°C and the cell pellets were stored at −80°C.

Cell pellets were resuspended in buffer A (20 mM HEPES, pH 7.5, 500 mM NaCl, 0.01% Tween-20 and 10 mM Imidazole) supplemented with 1 mM PMSF and 2 mg/ml DNase I, lysed by sonication and the lysate was clarified by centrifugation at 20,000 rpm for 1 h 30 min at 4°C.

The supernatant was loaded onto a 5 ml Ni-NTA His-Trap FF crude column (GE Healthcare), pre-equilibrated with buffer A. Unbound proteins were washed away with five column volumes of buffer B containing all components of buffer A, but 30 mM imidazole instead of 10 mM, and the protein was eluted with buffer C (buffer A plus 250 mM Imidazole). Fractions containing the desired protein were detected by using Bradford reagent for qualitative measurement that were later pooled and dialyzed overnight against buffer D (20 mM HEPES, pH 7.5, 0.5 M NaCl, 0.01% Tween-20) together with 3C protease (1 mg of 3C protease for 50 mg of protein) and 3 mM DTT to remove the hexa-histidine tag.

Uncleaved protein and protease were removed by running the sample through a Ni-NTA HisTrap column for a second time. The cleaved protein, which did not bind to the column material, was pooled and the buffer was exchanged with buffer E (50 mM HEPES, pH 7.5 and 0.5 M NaCl). All stages of protein purification were analysed by running samples on SDS-PAGE. The obtained protein was concentrated by ultrafiltration using a Centricon 30 kDa MWCO (Millipore) to reach a final concentration of 10 mg/ml. Finally, the protein was aliquoted in 50 µl aliquots, frozen in liquid nitrogen and stored at −80°C to be used for subsequent crystallization.

The same protocol was followed for DENV RdRp serotype 2 purification, but the following buffers were used: Buffer A1 (50 mM Tris, pH 8.8, 500 mM NaCl, 0.01% Tween-20 and 10 mM Imidazole), buffer B1 (50 mM Tris, pH 8.8, 500 mM NaCl, 0.01% Tween-20 and 30 mM Imidazole), buffer C1 (50 mM Tris, pH 8.8, 500 mM NaCl, 0.01% Tween-20 and 250 mM Imidazole) for affinity purification. Buffer D1 (50 mM Tris, pH 8.8, 0.5 M NaCl, 0.01% Tween-20) was used for overnight dialysis and cleavage with 3C-protease and was buffer-exchanged into buffer E1 (50 mM Tris, pH 8.8 and 0.5 M NaCl) for setting up crystallization trails.

### Protein crystallization trials, optimization for diffraction measurements

Crystallization trails were carried using the newly developed RdRp screen with RdRp serotype 3 at 10 mg/ml using a Mosquito Crystal (ttp Labtech) nano-drop robot and 96-well 3-drop Swissci plates (Molecular Dimensions) applying the vapour diffusion sitting drop method.

For the initial screen an equal volume of protein sample and well solution were mixed (100 nl:100 nl). Plates were then incubated at 291K (18°C) for several weeks with regular visual examination. Crystals were obtained in most of the drops within 2–4 days.

Diffraction quality single crystals for each successful condition were obtained using Crystalgen SuperClear™ Plates pre-greased 24-well linbro plates (Jena Biosciences). The drops were set up using 1 μl of protein (10 mg/ml) and 1 μl of well solution using the hanging drop vapour diffusion method.

Crystallization trials for RdRp serotype 2 were set up using the screen by mixing an equal volume of protein sample and well solution (e.g. 100 nl:100 nl). Plates were then incubated at two distinct temperatures at 277K (4°C) and 291K (18°C) for several weeks with regular visual examination. Crystals were obtained in most of the drops within 2–4 days at 18°C and after about 2 weeks at 4°C.

To obtain a co-crystal structure of RdRp serotype 3 in complex with PC-79-SH52 (Noble et al., 2016) using co-crystallization, the protein was incubated with 1 mM of the inhibitor for 1 h at 4°C before setting up crystallization drops.

### Determination of inhibitor soaking conditions for optimal diffraction measurements for RdRp serotype 3-inhibitor complexes

The RdRp serotype 3 inhibitor was provided as a 50 mM DMSO stock. For soaking experiments, we initially wanted to obtain the optimal DMSO concentrations and soaking times required without affecting the diffraction quality of our crystals. The graph in Fig. 4A shows how the diffraction quality varies with different DMSO concentrations present in the soaking solutions for different lengths of the soaking time. We determined soaking crystals with compound PC-79-SH52 at 10% DMSO for 1 h to be optimal. To increase our chances in obtaining RdRp3-inhibitor complex via soaking of native crystals with the inhibitor, we tested two different final concentrations of the inhibitor at 20 and 40 mM (maintaining 10% overall DMSO concentration).

For data collection, crystals were frozen in the presence of DMSO, which acted as a cryoprotectant. The crystals were then flash frozen in liquid nitrogen for subsequent measurements using synchrotron radiation.

### Data collection, structure determination

Diffraction data for each individual crystal were collected on Massif beamline ID30a-1 at the ESRF and at beamlines I03 and I04 at Diamond Light Source. Data were processed using either XDS (Kabsch, 2010) or iMosflm (Battye et al., 2011) and scaled to resolutions as mentioned in Table S4 (Winn et al., 2011). The structure of the dengue RdRp serotype 3 was solved by molecular replacement. Further details are provided in the section 3.4.2 and the crystallographic statistics are given in Table S5.

## Supplementary Material

Supplementary information
