# Supplementary material for: Development and validation of RdRp Screen, a crystallization screen for viral RNA-dependent RNA polymerases
Source: Biol Open. 2019 Jan 15;8(1):bio037663. doi: 10.1242/bio.037663 (PMC6361211; doi:10.1242/bio.037663)
Supplement: Supplementary information [file biolopen-8-037663-s1.pdf]

Supporting information

Development and validation of RDRP screen, a crystallization screen for viral RNA-dependent RNA polymerases

Federica Riccio[a1], Sandeep K. Talapatra[a1], Michela Mazzon[b] and Frank Kozielski[a]*

[a]Department of Pharmaceutical and Biological Chemistry, UCL School of Pharmacy, 29-39 Brunswick Square, London, WC1N 1AX, United Kingdom

[b]UCL MRC Laboratory for Molecular Cell Biology, Gower Street, London, WC1E 6BT, United Kingdom

Correspondence email: f.kozielski@ucl.ac.uk; s.talapatra@ucl.ac.uk

[1]Equal Contribution

S1. Summary

Figure S1: The Ligplot of the ligand binding pocket in the palm domain. Dashed green lines indicate hydrogen bonds, and the half-moon represents hydrophobic interactions between residues of the RdRp and compound PC-79-SH52.

Table S1: Data mining for RdRp crystallization conditions……………………………………..2

Table S2: The calculated volume of each stock solution used in each condition of the screen for setting up the crystallization screen……………………………………………………….……………..33

Table S3: Details of each chemical used in the screen composition…………………………………37

Table S4: Summary of approximate diffraction limits obtained for Dengue RdRp3 crystals in various well conditions of the crystallization screen..……………………………………………...……39

Table S5: Data collection and structure refinement statistics of dengue RdRp3 structures obtained from different crystallization conditions in the RdRp Screen……………………………………..40

Table S6: Details of missing regions, PEG and water molecules in the structure of Dengue RdRp3 solved………………..…………………………………………………………………………41

Biology Open • Supplementary information

Figure S1:

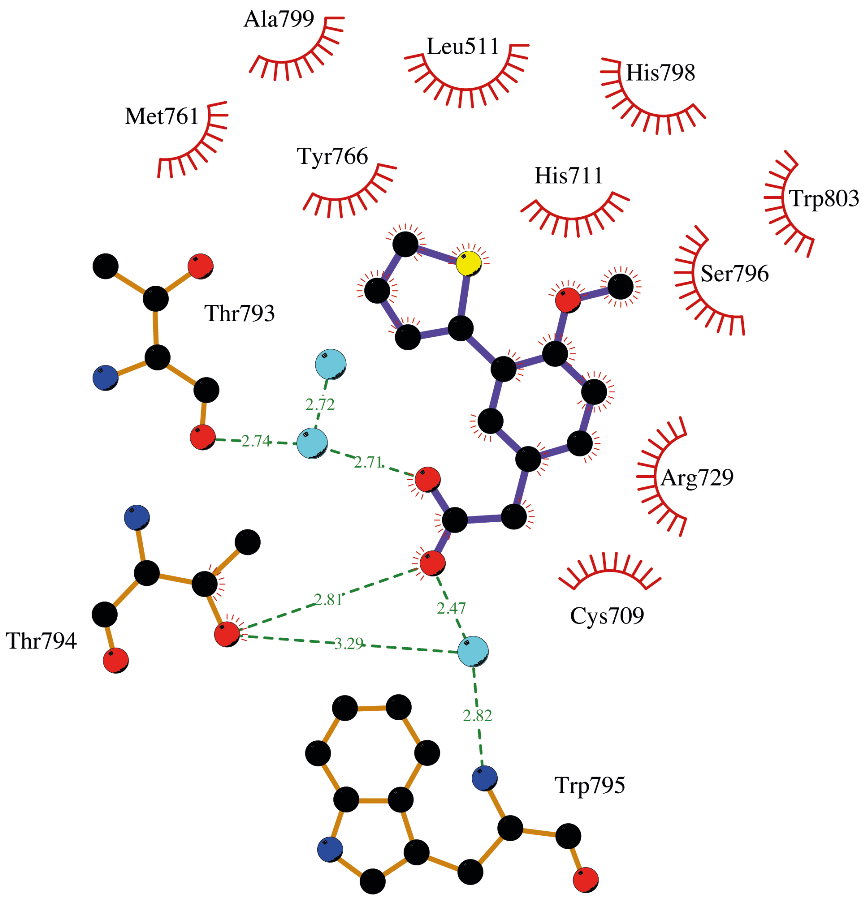

Biology Open • Supplementary information

Table S1 Data mining for RdRp crystallization conditions. The details of each RdRp structure submitted to the PDB were individually extracted to design the RdRp screen.

N°	Organism	Protein	PDB Id	Resolution (Å)	Space group	Method & Temperature (°C)	Crystallization condition	PMID & references
1	Bovine viral diarrhea virus	RdRp	2CJQ	2.60	$P3_1$	Sitting drop	10% PEG 8000<br>0.1 M HEPES, pH 7.5<br>8% Ethylene Glycol	(Choi *et al.*, 2006)
2	Coxsackievirus	B3 Polymerase F232L mutant	4WFX	1.81	$P4_32_12$	(16°C)	0.085 M HEPES,<br>3.655 M NaCl,<br>15% Glycerol	(Campagnola *et al.*, 2015)
3	Coxsackievirus	B3 Polymerase F232L mutant	4WFY	2.06	$P4_32_12$	(16°C)	0.085 M Tris, pH 7.5,<br>1.275 M $(NH_4)_2SO_4$,<br>25% Glycerol	
4	Coxsackievirus	B3 3D pol RdRp	4WFZ	1.80	$P4_32_12$	(16°C)	0.085 M HEPES, pH 7.5<br>3.6 M NaCl, 15% Glycerol	
5	Coxsackievirus	B3 3D pol with GPC-N114 inhibitor	4Y2A	2.90	$P4_32_12$	Sitting drop (20°C)	50 mM Tris, pH 7.5<br>24.5% Glycerol<br>1.29 M $(NH_4)_2SO_4$	(van der Linden *et al.*, 2015)
6	Coxsackievirus	B3 3D pol in	4Y34	2.70	$P4_32_12$	Sitting drop (20°C)	50 mM Tris pH 7.5	

Biology Open • Supplementary information

		complex with GPC-N143					24.5% Glycerol<br>1.2 9 M $(NH_4)_2SO_4$	
7	Coxsackievirus	B3 RdRp (3D pol) with pyrophosphate	3CDU	2.10	$P4_32_12$	Hanging drop (20°C)	2 M $(NH_4)_2SO_4$,<br>0.1 M CAPS, pH 10.0,<br>0.2 M Li_2SO_4	(Gruez *et al.*, 2008)
8	Coxsackievirus	B3 RdRp (3D pol) with protein primer VPg and a pyrophosphate	3CDW	2.50	$P4_32_12$	Hanging drop (20°C)	2 M $(NH_4)_2SO_4$,<br>0.1 M CAPS, pH 10.0<br>0.2 M Li_2SO_4	
9	Dengue virus serotype 3	NS5 RdRp	2J7U	1.85	$C222_1$	Hanging drop (4°C)	0.1 M Tris-HCl, pH 8.5<br>0.8 M K/Na Tartrate<br>0.5% PEG 5000 MME	(Yap *et al.*, 2007)
10	Dengue virus serotype 3	NS5 RdRp complexed with 3'DGTP	2J7W	2.60	$C222_1$	Hanging drop (4°C)	0.1 M Tris-HCl, pH 8.5<br>0.8 M K/Na Tartrate,<br>0.5% PEG 5000 MME	
11	Dengue virus serotype 3	RdRp	4HHJ	1.79	$C222_1$	Hanging drop (18°C)	0.1 M Tris, pH 8.0<br>25% PEG 550 MME	(Noble *et al.*, 2013)
12	Dengue virus serotype 3	RdRp bound to NITD-107	3VWS	2.10	$C222_1$	Hanging drop (18°C)	20% PEG 550 MME<br>0.1 M Tris pH 8.0	

13	Dengue virus serotype 3	RdRp with residues from the NS5 linker region	4C11	2.60	$P2_12_12$	Sitting drop (20°C)	1.0 M Succinic Acid, 0.1 M HEPES, pH 7.0 1% PEG 2000 MME	(Lim *et al.*, 2013)
14	Dengue virus serotype 3	RdRp bound to PC-79-SH52	5F3Z	2.00	$C222_1$	Hanging drop (18°C)	0.1 M Tris, pH 8.0 25% PEG 550 MME	(Noble *et al.*, 2016)
15	Dengue virus serotype 3	RdRp bound to FD-83-ki26	5F41	2.00	$C222_1$	Hanging drop (18°C)		
16	Dengue virus serotype 3	RdRp bound to JF-31-MG46	5F3T	2.05	$C222_1$	Hanging drop (18°C)		
17	Dengue virus serotype 3	NS5 full length with SAH	4V0Q	2.30	$P2_12_12$	Sitting drop (20°C)	0.2 M $CaAc_2$ or $MgAc_2$ 0.1 M Na Cacodylate, pH 6.4 10-20% PEG 8000	(Zhao *et al.*, 2015)
18	Dengue virus serotype 3	NS5 full length with GTP and SAH	4V0R	2.40	$P2_12_12$	Sitting drop (18°C)	0.1 M Na Cacodylate, pH 6.4 0.2 M $MgAc_2$ or $CaAc_2$ 14% PEG 8000	
19	Enterovirus 71	RdRp	3N6L	2.60	$P3_22_1$	Hanging drop (18°C)	1.3 M $(NH_4)_2SO_4$	(Wu *et al.*, 2010)
20	Enterovirus 71	RdRp	3N6M	2.50	$P3_22_1$	Hanging drop (18°C)	1 mM DTT	

21	Enterovirus 71	RdRp	3N6N	2.90	$P3_22_1$	Hanging drop (18°C)	0.1 M Bis-Tris pH 6.1<br>10 mM $NiCl_2$	
22	Foot and mouth disease virus	RdRp K18E mutant with RNA	4WZM	2.57	$P3_22_1$	Hanging drop (20°C)	30% PEG 4000<br>0.2 M $MgAc_2$,<br>0.1 M MES pH 6.0<br>4% γ-butyrolactone	(Ferrer-Orta *et al.*, 2015)
23	Foot and mouth disease virus	3D pol K18E mutant	4WYL	2.00	$P4_12_12$	Hanging drop (20°C)		
24	Foot and mouth disease virus	3D pol K20E mutant	4WYW	1.80	$P4_12_12$	Hanging drop (20°C)		
25	Foot and mouth disease virus	RdRp K20E mutant with RNA	4WZQ	2.80	$P3_22_1$	Hanging drop (20°C)	30% PEG 4000<br>0.2 M $MgAc_2$<br>0.1 M MES pH 6.0	
26	Foot and mouth disease virus	RdRp K20A mutant with an RNA	4X2B	2.94	$P4_12_12$	Hanging drop (20°C)		
27	Foot and mouth disease virus	RdRp with a template-primer RNA and ribavirin	2E9R	2.81	$P3_22_1$	Hanging drop (20°C)	30% PEG 4000<br>0.2 M $MgAc_2$<br>0.1 M Na Cacodylate pH 6.0	(Ferrer-Orta *et al.*, 2007)
28	Foot and mouth disease virus	RdRp with a template-	2E9T	2.60	$P3_22_1$	Hanging drop (20°C)		

		primer RNA and ATP						
29	Foot and mouth disease virus	RdRp with a template-primer RNA and 5F-UTP	2EC0	2.70	$P3_22_1$	Hanging drop (20°C)	33% NH_4Ac<br>0.1 M Na Citrate pH 5.6<br>4% butyrolactone	
30	Foot and mouth disease virus	RdRp with uridylylated VPg protein	2F8E	2.90	$P3_22_1$	Hanging drop (20°C)	33% PEG 4000<br>0.2 M NH_4Ac<br>0.1 M Na Citrate, pH 5.6<br>4% Butyrolactone	(Ferrer-Orta *et al.*, 2006)
31	Foot and mouth disease virus	RdRp with VPg protein	2D7S	3.00	$P3_22_1$	Hanging drop (20°C)		
32	Hepatitis C virus	NS5B RdRp	1C2P	1.90	$P2_12_12_1$	Batch (22°C)	10% PEG 4000, 10% Glycerol, 5 mM DTT, 5 mM Tris, pH 7.5,<br>25 mM MES, pH 5.0	(Lesburg *et al.*, 1999)
33	Hepatitis C virus	RdRp	1CSJ	2.80	$P2_12_12_1$	Hanging drop (4°C)	5% PEG 8000, 5% 2-propanol<br>0.1 M Na Citrate, pH 6.5	
34	Hepatitis C virus	RNA pol in complex with GTP and Mn^{2+}	1GX5	1.70	$P2_12_12_1$	Hanging drop (4°C)	4% PEG 4000<br>7% 2-Propanol<br>0.1 M Na Citrate, pH 6.8	(Bressanelli *et al.*, 2002)
35	Hepatitis C virus	RNA pol in complex with	1GX6	1.85	$P2_12_12_1$	Hanging drop (4°C)		

Biology Open • Supplementary information

		UTP and Mn^{2+}						
36	Hepatitis C virus	RNA pol with non-nucleoside analogue inhibitor	1NHU	2.00	$P2_12_12_1$	Hanging drop (22°C)	18% PEG 4000<br>0.3 M NaCl<br>0.1 M NaOAc, pH 5.0,<br>0 5 mM 2-mercaptoethanol	(Wang *et al.*, 2003)
37	Hepatitis C virus	RNA pol with NNI	1NHV	2.90	$P2_12_12_1$	Hanging drop (22°C)		
38	Hepatitis C virus	NS5B RNA pol with non-competitive inhibitor	1OS5	2.20	$P4_12_12$	Hanging drop (20°C)	0.2 M NH_4Ac, pH 4.6<br>30% PEG MME 2000	(Love *et al.*, 2003)
39	Hepatitis C virus	RdRp	1QUV	2.50	$P4_32_12$	Hanging drop (22.5°C)	21 – 28% PEG 4000, 0.2 -0.35 M NaOAc, 0.1M NH_4Ac<br>0.02 M TES, pH 6.0	(Ago *et al.*, 1999)
40	Hepatitis C virus	NS5b RNA Pol with a covalent inhibitor	2AWZ	2.15	$P2_12_12_1$	Hanging drop (20°C)	200 mM $(NH_4)_2SO_4$<br>27% PEG 5000 MME<br>100 mM NaOAc, pH 5.0	(Powers *et al.*, 2006)
41	Hepatitis C virus	NS5b Pol with a covalent	2AX1	2.10	$P2_12_12_1$	Hanging drop (20°C)		

Biology Open • Supplementary information

		inhibitor						
42	Hepatitis C virus	Pol with an allosteric inhibitor	2BRK	2.30	$P2_12_12$	Hanging drop (4°C)	5% PEG 8000<br>5% 2-propanol<br>0.1 M Na Citrate, pH 6.5	(Di Marco *et al.*, 2005)
43	Hepatitis C virus	Pol with an allosteric inhibitor	2BRL	2.30	$P2_12_12$	Hanging drop (4°C)		
44	Hepatitis C virus	NS5B pol with a tetracyclic inhibitor	2DXS	2.20	P_12_1	Hanging drop (4°C)	0.1 M Citrate, pH 5.5<br>8% PEG 8000<br>5% 2-propanol	(Ikegashira *et al.*, 2006)
45	Hepatitis C virus	NS5B RdRp with NNI-2 inhibitor	2GIQ	1.65	$P2_12_12_1$	Hanging drop (20°C)	50 mM Na Citrate pH 4.9<br>26% PEG 4000<br>7.5% Glycerol	(Le Pogam *et al.*, 2006)
46	Hepatitis C virus	NS5B RdRp with NNI-1 inhibitor	2GIR	1.90	$P2_12_12_1$	Hanging drop (20°C)		
47	Hepatitis C virus	NS5B pol with dihydropyrone -containing inhibitor	2HAI	1.58	$P4_12_12$	Hanging drop (20°C)	30% PEG MME 2000,<br>0.2 M $(NH_4)_2SO_4$<br>0.1 M NH_4Ac, pH 5.0	(Li *et al.*, 2006)
48	Hepatitis C virus	NS5B pol with Thiazolones	2I1R	2.20	$P2_12_12_1$	Evaporation (22°C)	20% PEG 400<br>5 mM DTT	(Yan, Larson, *et al.*, 2007)

		inhibitor					0.5 M MES, pH 5.0<br>1.0 M NaCl	
49	Hepatitis C virus	NS5B polymerase	2IJN	2.20	$P2_12_12_1$	Evaporation (22°C)	20% PEG 4000<br>5 mM DTT<br>0.5 M MES, pH 5.0<br>1.0 M NaCl	(Yan, Appleby, Gunic, *et al.*, 2007)
50	Hepatitis C virus	NS5B with allosteric inhibitor	2HWH	2.30	$P2_12_12_1$	Evaporation (22°C)	20% PEG 4000<br>5 mM DTT<br>0.5 M MES, pH 5.0<br>1.0 M NaCl	(Yan *et al.*, 2006)
51	Hepatisis C virus	NS5B with allosteric inhibitor	2HWI	2.00	$P2_12_12_1$	Evaporation (22°C)		
52	Hepatisis C virus	Polymerase with inhibitor SB655264	2JC0	2.00	$P2_12_12_1$		20% PEG 4000,<br>5 mM DTT,<br>10% Glycerol<br>0.1 M Citrate buffer, pH 5.0	(Slater *et al.*, 2007)
53	Hepatisis C virus	Polymerase with inhibitor SB698223	2JC1	2.00	$P2_12_12_1$			
54	Hepatisis C virus	NS5B pol with Thiazolone-acylsulfonamides allosteric	2O5D	2.20	$P2_12_12_1$	Evaporation (22°C)	20% PEG 4000<br>5 mM DTT<br>0.5 M MES, pH 5.0	(Yan, Appleby, Larson, *et al.*, 2007)

Biology Open • Supplementary information

		inhibitors					1.0 M NaCl	
55	Hepatitis C virus – H77	NS5B apo polymerase	2XI2	1.80	P 2_1	Sitting drop	0.1 mM MES, pH 6.5<br>0.2 M $(NH_4)_2SO_4$<br>PEG 5000 MME	(Harrus *et al.*, 2010)
56	Hepatitis C virus – H77	NS5B polymerase with GTP	2XI3	1.70	P1	Sitting drop	0.05 M MES, pH 6.5, 0.2 M $(NH_4)_2SO_4$<br>0.25 M NH_4Ac<br>25%-30% PEG 1000	
57	Hepatitis C virus	NS5B pol with Thienopyrrole -based finger-loop inhibitors	2WCX	2.00	$P2_12_12$		0.1 M MES, pH 6.0<br>14% PEG 8000<br>14% 2-propanol<br>10 mM DTT,<br>10 mM $MnCl_2$	(Martin Hernando *et al.*, 2009)
58	Hepatitis C virus genotype 1B	NS5B pol with a NNI	2WHO	2.00	$P2_12_12_1$		0.1 M MES, pH 6.0<br>14% PEG 8000<br>14% 2-propanol<br>10 mM DTT<br>10 mM $MnCl_2$	(Ontoria *et al.*, 2009)
59	Hepatitis C virus	NS5B pol with an allosteric	2WRM	1.95	$P2_12_12$		pH 6.0	To be published

Biology Open • Supplementary information

		inhibitor of thumb domain						
60	Hepatitis C virus	NS5B pol with a potent non-nucleoside finger-loop inhibitor	2XWY	2.53	$P2_12_12$		0.1 M MES, pH 6.0<br>14% PEG 8000<br>2.5 mM TCEP<br>14% 2- propanol<br>10 mM $MnCl_2$	(Narjes *et al.*, 2011)
61	Hepatitis C virus	NS5B polymerase	2ZKU	1.95	$P2_12_12_1$	Hanging drop (25°C)	5 mM 2- Mercaptoethanol<br>18% PEG 4000<br>0.3 M NaCl<br>0.1 M NaOAc, pH 5.0	
62	Hepatitis C virus strain JFH1	NS5B polymerase	3I5K	1.90	$P2_1$	Hanging drop (20°C)	6 to 7% PEG 20000<br>0.2 M NaH_2PO4, pH 7.0	(Simister *et al.*, 2009)
63	Hepatitis C virus	NS5B pol with a novel Pyridazinone inhibitor	3BR9	2.30	$P2_12_12_1$	Hanging drop (25°C)	20% PEG 4000<br>5 mM DTT<br>50 mM $(NH_4)_2SO_4$<br>0.1 M NaOAc, pH 7.4	(Zhou *et al.*, 2008)
64	Hepatitis C virus	NS5B pol with a novel Pyridazinone	3BSA	2.30	$P2_12_12_1$	Hanging drop (25°C)		

Biology Open • Supplementary information

		inhibitor						
65	Hepatitis C virus	NS5B pol with a novel Pyridazinone inhibitor	3BSC	2.65	$P2_12_12_1$	Hanging drop (25°C)		
66	Hepatitis C virus	NS5B pol with a novel Pyridazinone inhibitor	3CDE	2.10	$P2_12_12_1$	Hanging drop (25°C)	20% PEG 4000<br>5 mM DTT<br>50 mM $(NH_4)_2SO_4$<br>0.1 M NaOAc	(Li *et al.*, 2008)
67	Hepatitis C virus	NS5B RdRp with small molecule fragments	3CIZ	1.87	$P2_12_12_1$	Hanging drop (19°C)	8-11% Glycerol<br>20-24% PEG 4000<br>50 mM Na Citrate, pH 4.9	(Antonysamy *et al.*, 2008)
68	Hepatitis C virus	NS5B RdRp with small molecule fragments	3CJ0	1.90	$P2_12_12_1$	(19°C)		
69	Hepatitis C virus	NS5B RdRp with small molecule fragments	3CJ2	1.75	$P2_1$	(19°C)		
70	Hepatitis C virus	NS5B RdRp	3CJ3	1.87	$P2_12_12_1$	(19°C)		

Biology Open • Supplementary information

		with small molecule fragments						
71	Hepatitis C virus	NS5B RdRp with small molecule fragments	3CJ4	2.07	$P2_1$	(19°C)		
72	Hepatitis C virus	NS5B RdRp with small molecule fragments	3CJ5	1.92	$P2_12_12_1$	(19°C)		
73	Hepatitis C virus	NS5B pol with a novel Pyridazinone inhibitor	3CO9	2.10	$P2_12_12_1$	(25°C)	20% PEG 4000<br>5 mM DTT<br>50 mM $(NH_4)_2SO_4$<br>0.1 M NaOAc, pH 4.7	(Ruebsam *et al.*, 2008)
74	Hepatitis C virus	NS5B pol with a novel Pyridazinone inhibitor	3CVK	2.31	$P2_12_12_1$	Hanging drop (25°C)	20% PEG 4000<br>5 mM DTT<br>50 mM $(NH_4)_2SO_4$<br>0.1 M NaOAc, pH 4.7	(Ellis *et al.*, 2008)
75	Hepatitis C virus	NS5B pol with a novel Pyridazinone	3CWJ	2.40	$P2_12_12_1$	Hanging drop (25°C)	20% PEG 4000<br>5 mM DTT	(Ellis *et al.*, 2008)

Biology Open • Supplementary information

		inhibitor					50 mM $(NH_4)_2SO_4$<br>0.1 M NaOAc, pH 4.7	
76	Hepatitis C virus	NS5B pol with a novel Benziothiazole inhibitor	3D28	2.30	$P2_12_12_1$	Hanging drop (25°C)	20% PEG 4000<br>5 mM DTT<br>50 mM $(NH_4)_2SO_4$<br>0.1 M NaOAc, pH 4.7	(Kim *et al.*, 2008)
77	Hepatitis C virus	NS5B pol with a novel Pyridazinone inhibitor	3D5M	2.20	$P2_12_12_1$	Hanging drop (25°C)		
78	Hepatitis C virus	NS5B pol with a novel Pyridazinone inhibitor	3E51	1.90	$P2_12_12_1$	Hanging drop (25°C)	20% PEG 4000<br>5 mM DTT<br>50 mM $(NH_4)_2SO_4$<br>0.1 M NaOAc, pH 4.7	(Dragovich *et al.*, 2008)
79	Hepatitis C virus	NS5B pol with 796 inhibitor	3FQK	2.20	$P2_12_12_1$	Hanging drop (20°C)	50 mM Na Citrate, pH 4.9<br>7.5% Glycerol<br>24% PEG 4000	(Hang *et al.*, 2009)
80	Hepatitis C virus	NS5B pol with 796 inhibitor	3FQL	1.80	$P2_12_12_1$	Sitting drop (20°C)	50 mM Na Citrate pH 4.9<br>7.5% Glycerol<br>26% PEG 4000	

81	Hepatitis C virus	NS5B RNA pol with PF868554	3FRZ	1.86	$P4_12_12$	Hanging drop (25°C)	18.0% PEG 3000<br>14% Glycerol<br>0.1 M Citrate, pH 5.5	(Li *et al.*, 2009)
82	Hepatitis C virus	NS5B pol with thiazine inhibitor	3G86	2.20	$P2_12_12_1$	Sitting drop	50 mM Na Citrate pH 4.9<br>7.5% Glycerol<br>24% PEG 4000	(de Vicente *et al.*, 2009)
83	Hepatitis C virus	NS5B pol with a monocyclic dihydro-pyridinone inhibitor	3GYN	2.15	$P2_12_12_1$	Hanging drop (25°C)	20% PEG 4000<br>5 mM DTT<br>50 mM $(NH_4)_2SO_4$<br>0.1 M NaOAc, pH 4.7	(Ellis *et al.*, 2009)
84	Hepatitis C virus	NS5B pol with a monocyclic dihydro-pyridinone inhibitor	3IGV	2.60	$P2_12_12_1$	Hanging drop (25°C)		
85	Hepatitis C virus	NS5B pol with a bicyclic dihydro-pyridinone inhibitor	3H2L	1.90	$P2_12_12_1$	Hanging drop (25°C)	20% PEG 4000<br>5 mM DTT<br>50 mM $(NH_4)_2SO_4$<br>0.1 M NaOAc, pH 4.7	(Ruebsam *et al.*, 2009)
86	Hepatitis C virus	NS5B pol	3H59	2.10	$P2_12_12_1$	Hanging drop (20°C)	50 mM Na Citrate pH 4.9	

Biology Open • Supplementary information

		thiazine inhibitor 2					26% PEG 4000<br>7.5% Glycerol	(de Vicente, *et al.*, 2009)
87	Hepatitis C virus	NS5B pol with saccharin inhibitor	3H5S	2.00	$P2_12_12_1$	Sitting drop (20°C)	50 mM Na Citrate, pH 4.9<br>26% PEG 4000<br>7.5% Glycerol	
88	Hepatitis C virus	NS5B pol with saccharin inhibitor 1	3H5U	1.95	$P2_12_12_1$	Sitting drop (20°C)	50 mM Na Citrate, pH 4.9<br>24% PEG 3350<br>7.5% Glycerol	
89	Hepatitis C virus	NS5b 1b with a pyrimidine derivative	3H398	1.90	$P2_12_12_1$	Sitting drop (20°C)	12-17.5% PEG 4000<br>8-11% Glycerol<br>0.1 M Na Citrate, pH 4.5-6.0	(Wang *et al.*, 2009)
90	Hepatitis C virus	NS5B pol with thumb site inhibitor	3MF5	2.00	$P2_12_12_1$	Sitting drop (0°C)	26% PEG 4000<br>7.5% Glycerol<br>50 mM Na Citrate, pH 4.9	(Yang *et al.*, 2010)
91	Hepatitis C virus	NS5B pol with 3-heterocyclic quinolone	3UDL	2.15	$P2_12_12_1$	(18°C)	16% PEG 4000<br>10% Glycerol<br>0.3 M NaCl<br>5 mM ß-mercaptoethanol<br>0.1 M NaOAc, pH 5.1	(Kumar *et al.*, 2011)

Biology Open • Supplementary information

92	Hepatitis C virus	NS5B RNA pol with a piperazine inhibitor	3QVS	1.90	$P2_12_12_1$	Hanging drop (10°C)	0.1 M NaOAc, pH 6.0<br>14% PEG 4000<br>0.3 M NaCl<br>5 mM 2-mercaptoehanol<br>2.4 mM n-octanoylsucrose	(Ando *et al.*, 2012)
93	Hepatitis C virus	NS5B genotype 2A JFH-1 with primer-template RNA	4E7A	3.00	$P6_5$	Sitting drop (16°C)	30% PEG 3350<br>0.1 M Bis-Tris propane, pH 6.0<br>0.2 M NH_4Ac	(Mosley *et al.*, 2012)
94	Hepatitis C virus	NS5B genotype 2A JFH-1 with primer template RNA	4E78	2.50	$P6_5$	Sitting drop (16°C)		
95	Hepatitis C virus	NS5B genotype 2A JFH-1 with beta hairpin loop deletion	4E76	2.90	$P6_5$	Sitting drop (16°C)	30% PEG 550 MME<br>0.1 M Bis-Tris propane, pH 6.5<br>50 mM $(NH_4)_2SO_4$	
96	Hepatitis C virus	NS5B pol inhibited by Tri-substituted	4EO6	1.79	$P2_12_12_1$	Sitting drop (20°C)	15-20% PEG 4000<br>10% Glycerol,	(Canales *et al.*, 2012)

Biology Open • Supplementary information

		acylhydrazines					0.1 M NaOAc, pH 4.8-5.0	
97	Hepatitis C virus	NS5B pol inhibited by Tri-substituted acylhydrazines	4EO8	1.80	$P2_12_12_1$	Sitting drop (20°C)		
98	Hepatitis C virus	NS5B (BK)pol with fragment-based compounds	4IH5	1.90	$P2_12_12_1$	Sitting drop (25°C)	26% PEG 4000<br>7.5% Glycerol<br>50 mM Na Citrate, pH 4.9	(Talamas *et al.*, 2013)
99	Hepatitis C virus	NS5B (BK)pol with fragment-based compounds	4IH6	2.20	$P2_1$	Sitting drop (20°C)		
100	Hepatitis C virus	NS5B (BK)pol with fragment-based compounds	4IH7	2.30	$P2_12_12_1$	Sitting drop (25°C)		
101	Hepatitis C virus	NS5B GT1B N316 with GSK5852A	4KAI	2.19	$P2_12_12_1$	Sitting drop (25°C)	0.1 M Na-citrate pH 5.0<br>17% PEG 4000	(Maynard *et al.*, 2014)

Biology Open • Supplementary information

102	Hepatitis C virus	NS5B GT1B N316Y with CMPD 32	4KB7	1.81	$P2_12_12_1$	Sitting drop (25°C)	10% Glycerol	
103	Hepatitis C virus	NS5B GT1B N316Y with CMPD 4	4KBI	2.06	$P2_12_12_1$	Sitting drop (25°C)		
104	Hepatitis C virus	NS5B GT1B N316Y with GSK5852	4KE5	2.10	$P2_12_12_1$	Sitting drop (25°C)		
105	Hepatitis C virus	NS5B 1b (BK) with RG7109	4MIA	2.80	$P2_12_12_1$	Sitting drop (19°C)	24% PEG 4000<br>7.5% Glycerol<br>50 mM Na Citrate, pH 4.6	(Talamas *et al.*, 2014)
106	Hepatitis C virus	NS5B 1b (BK) with Compound 48	4MIB	2.30	$P2_12_12_1$	Sitting drop (19°C)	26% PEG 4000<br>7.5% Glycerol<br>50 mM Na Citrate, pH 4.9	
107	Hepatitis C virus	NS5B 1b (BK) with inhibitor 2	4MK7	2.80	$P2_12_12_1$	Sitting drop (19°C)	26% PEG 4000<br>7.5% Glycerol<br>50 mM Na Citrate, pH 4.9	(Schoenfeld *et al.*, 2013)
108	Hepatitis C virus	NS5B 1b (BK) with inhibitor 4	4MK8	2.09	$P2_12_12_1$	Sitting drop (19°C)		

Biology Open • Supplementary information

109	Hepatitis C virus	NS5B 1b (BK) with inhibitor 14	4MKB	1.90	$P2_12_12_1$	Sitting drop (19°C)		
110	Hepatitis C virus	NS5B 1b (BK) with inhibitor 12	4MK9	2.05	$P2_12_12_1$	Sitting drop (19°C)	26% PEG 4000<br>7.5% Glycerol<br>50 mM Tris, pH 7.5	
111	Hepatitis C virus	NS5B 1b (BK) with inhibitor 13	4MKA	2.05	$P2_12_12_1$	Sitting drop (19°C)		
112	Hepatitis C virus	NS5B 2A with S15G, C223H, V321I mutations	4OBC	2.50	$P6_5$	Sitting drop (16°C)	PACT screen condition D5:<br>25% PEG 1500<br>0.1 M MMT buffer, pH 8.0	(Lam *et al.*, 2014)
113	Hepatitis C virus – J6	NS5B pol V405I mutant	4ADP	1.90	$P2_12_12_1$		17% PEG 4000<br>0.05 M Tri-Na Citrate, pH 6.5	(Scrima *et al.*, 2012)
114	Hepatitis C virus – J4	RNA pol W550N mutant	4RY5	2.71	$P2_12_12_1$	Hanging drop (18°C)	50 mM MES, pH 5.0<br>20% PEG 4000<br>5% Glycerol	(Cherry *et al.*, 2015)
115	Hepatitis C virus – J4	RNA pol W550A mutant	4RY6	2.52	$P2_12_12_1$	Hanging drop (18°C)		
116	Hepatitis C virus	RNA pol	4RY7	3.00	$P2_12_12_1$	Hanging drop (18°C)		

Biology Open • Supplementary information

	– J4	D559E mutant						
117	Hepatitis C virus	NS5B 2A JFH-1 with E86Q, E87Q, S15G, C223H, V321I and DELTA8 mutations (Apo)	4WT9	2.50	$P6_5$	Sitting drop (16°C)	30% PEG 550 MME<br>0.1 M HEPES, pH 7.5<br>50 mM $MgCl_2$	(Appleby *et al.*, 2015)
118	Hepatitis C virus	NS5B 2A JFH-1 with S15G, E86Q, C233H, V321I and DELTA8 mutations with UDP, Mn^{2+}	4WTA	2.80	$P6_5$	Sitting drop (16°C)	25% PEG 550 MME<br>0.1 M HEPES, pH 7.5<br>50 mM $MgCl_2$	
119	Hepatitis C virus	NS5B 2A JFH-1 with S15G, E86Q, C233H, V321I and DELTA8 mutations with CDP, Mn^{2+}	4WTC	2.75	$P6_5$	Sitting drop (16°C)		
120	Hepatitis C virus	NS5B 2A	4WTD	2.70	$P6_5$	Sitting drop (16°C)		

		JFH-1 with S15G, E86Q, C233H, V321I and DELTA8 mutations with ADP, Mg^{2+}				
121	Hepatitis C virus	NS5B 2A JFH-1 with S15G, E86Q, C233H, V321I and DELTA8 mutations with GDP, Mg^{2+}	4WTE	2.90	$P6_5$	Sitting drop (16°C)
122	Hepatitis C virus	NS5B 2A JFH-1 with S15G, E86Q, C233H, V321I and DELTA8 mutations with GS-639475, Mg^{2+}	4WTF	2.65	$P6_5$	Sitting drop (16°C)
123	Hepatitis C virus	NS5B 2A JFH-1 with S15G, E86Q,	4WTG	2.90	$P6_5$	Sitting drop (16°C)

Biology Open • Supplementary information

		C233H, V321I and DELTA8 mutations with GS-607596, Mg^{2+}				
124	Hepatitis C virus	NS5B 2A JFH-1 with S15G, E86Q, C233H, V321I mutations with RNA templates, Mg^{2+} GDP	4WTI	2.80	$P6_5$	Sitting drop (16°C)
125	Hepatitis C virus	NS5B 2A JFH-1 with S15G, E86Q, C233H, V321I mutations with RNA templates, Mg^{2+} ADP	4WTJ	2.20	$P6_5$	Sitting drop (16°C)
126	Hepatitis C virus	NS5B 2A JFH-1 with S15G, E86Q,	4WTK	2.50	$P6_5$	Sitting drop (16°C)

Biology Open • Supplementary information

		C233H, V321I mutations with RNA templates, Mg^{2+} CDP						
127	Hepatitis C virus	NS5B 2A JFH-1 with S15G, E86Q, C233H, V321I mutations with RNA templates, Mg^{2+} UDP	4WTL	2.00	$P6_5$	Sitting drop (16°C)		
128	Hepatitis C virus	NS5B 2A JFH-1 with S15G, E86Q, C233H, V321I mutations with RNA templates, Mg^{2+} UDP	4WTM	2.15	$P6_5$	Sitting drop (16°C)		
129	Hepatitis C Virus	RNA pol apo-form	1NB4	2.00	$P2_12_12_1$	(18°C)	50 mM MES, pH 5.0<br>20% PEG 4000	(O'Farrell *et al.*, 2003)
130	Hepatitis C Virus	RNA pol with	1NB6	2.60	$P2_12_12_1$	(18°C)		

Biology Open • Supplementary information

		UTP					10% Glycerol	
131	Hepatitis C Virus	RNA pol with short RNA template	1NB7	2.90	$P2_12_12_1$	(18°C)	5 mM DTT	
132	Human Norovirus	RdRp with inhibitor PPNDS	4LQ3	2.60	I222	Sitting drop (20°C)	1.2 M Na Citrate<br>0.125 M NaCl<br>0.1 M Na cacodylate	(Tarantino *et al.*, 2014)
133	Human Norovirus	RdRp in complex with NAF2	4LQ9	2.04	I222	Sitting drop (20°C)		
134	Human Norovirus	Pol bound to suramin derivative	4NRT	2.02	I222	Sitting drop (20°C)	1.2 M Na citrate,<br>0.125 M NaCl<br>0.1 M Na cacodylate, pH 6.2	(Croci, Pezzullo, *et al.*, 2014)
135	Human Rhinovirus 16	RdRp	1TP7	2.40	$P2_1$	Sitting drop (22°C)	40% PEG 4000<br>0.1 M Na citrate pH 5.6,<br>35 mM $(NH_4)_2SO_4$<br>5% Glycerol<br>5 mM DTT	(Appleby *et al.*, 2005)
136	Infectious bursal disease virus	RdRp VP1	2PGG	2.50	$P6_122$	Hanging drop (20°C)	0.1 M HEPES, pH 7.5<br>1.3 M Na malonate	(Pan *et al.*, 2007)

Biology Open • Supplementary information

							10 mM NaI	
137	Infectious bursal disease virus	VP1 pol with oligopeptide	2QJ1	3.48	$P6_122$	Hanging drop (20°C)	10-12% PEG 3350<br>0.3-0.5 M $LiNO_3$, pH 6.5-8.0	(Garriga *et al.*, 2007)
138	Infectious bursal disease virus	RdRp	2PUS	2.40	$P6_122$	Hanging drop (25°C)		
139	Infectious bursal disease virus	VP1 pol with oligopeptide	2R70	2.70	$P6_122$	Hanging drop (20°C)	5% PEG 3350<br>0.4 M $LiNO_3$<br>0.1 M Tris, pH 7.3	
140	Infectious bursal disease virus	VP1 pol with Mg^{2+} ion	2R72	3.15	$P6_122$	Hanging drop (20°C)	10% PEG 3350<br>0.4 M $LiNO_3$<br>0.1 M MES, pH 6.0	
141	Infectious Pancreatic Necrosis Virus	VP1-VP3 complex	3ZED	2.20	$P2_12_12$		20% (w/v) PEG 3350<br>0.2 M KF, pH 7.5	(Bahar *et al.*, 2013)
142	Infectious Pancreatic Necrosis Virus	RNA polymerase VP1	2YI8	2.30	$P2_12_12_1$	Sitting drop	18-20% PEG 3350<br>0.09-0.1 M Bis-Tris Propane pH 7.5	(Graham *et al.*, 2011)
143	Infectious Pancreatic Necrosis Virus	RNA polymerase VP1	2YI9	2.20	$P2_12_12_1$	Sitting drop	0.18-0.2 M Na Citrate	
144	Infectious	RNA	2YIA	3.02	$P2_12_12_1$	Sitting drop		

Biology Open • Supplementary information

	Pancreatic Necrosis Virus	polymerase VP1						
145	Infectious Pancreatic Necrosis Virus	RNA polymerase VP1	2YIB	3.80	$P2_12_12_1$	Sitting drop	20% PEG 3350<br>0.1 M Bis-Tris propane, pH 7.5<br>0.2 M Na Citrate<br>20 mM ATP<br>5% MPD<br>10 mM NaOH	
146	Influenza C virus	RdRp	5D9A	4.30	$P2_12_12_1$	Sitting drop (20°C)	0.2 M NaCl<br>25% PEG 4000<br>0.1 M HEPES, pH 7.5	(Hengrung *et al.*, 2015)
147	Influenza C virus	RdRp	5D98	3.90	$P4_32_12$	Sitting drop (20°C)	Morpheus condition G2:<br>10% PEG 8000,<br>20% ethylene glycol<br>0.02 M carboxylic acids<br>0.1 M MES/imidazole	
148	Japanese Encephalitis virus	RdRp	4MTP	3.65	$P2_12_12_1$	Hanging drop (25°C)	10-15% PEG 5000 MME<br>0.1 M Tris-HCl, pH 8.0<br>0.2 M NaSCN	(Surana *et al.*, 2014)

Biology Open • Supplementary information

149	Murine Norovirus	RdRp	3NAH	2.75	C2	Hanging drop	1 M $(NH_4)_2SO_4$	To Be Published
150	Murine Norovirus	RdRp	3NAI	2.55	C2	Hanging drop (4°C)	0.1 M Cacodylate pH 6.5	
151	Norwalk Virus	Polymerase (Triclinic)	1SH0	2.17	P1	Hanging drop (22°C)	24% PEG 8000<br>0.1-0.2 M $(NH_4)_2SO_4$	(Ng *et al.*, 2004)
152	Norwalk Virus	Polymerase (Metal-free, Centered Orthorhombic)	1SH2	2.30	$C222_1$	Hanging drop (22°C)	50 mM Tris-HCl, pH 7.5<br>15 % Glycerol<br>14 mM 2-mercaptoethanol	
153	Norwalk Virus	Polymerase (MgSO4 crystal form)	1SH3	2.95	$P2_12_12_1$	Hanging drop (22°C)		
154	Murine Norovirus	RdRp	3MWV	2.20	$P2_12_12_1$	(11°C)	0.1 M MES, pH 5.4<br>21% PEG 5000 MME<br>0.4 M $(NH_4)_2SO_4$<br>10% Glycerol	(LaPlante *et al.*, 2010)
155	Murine Norovirus	RdRp	3QID	2.50	C2	Hanging drop (22°C)	1.26 M $(NH_4)_2SO_4$<br>0.1 M Cacodylate pH 6.5	(Lee *et al.*, 2011)
156	Murine Norovirus	RdRp with 2 thiouridine (2TU)	3SFG	2.20	C2	Hanging drop (4°C)	2 M $(NH_4)_2SO_4$<br>Cacodylate pH 6.5<br>0.2 M NaCl	(Alam *et al.*, 2012)

Biology Open • Supplementary information

157	Murine Norovirus	RdRp with ribavirin	3SFU	2.50	C2	Hanging drop (20°C)	1.26 M $(NH_4)_2SO_4$<br>CHES, pH 9.5<br>0.2 M NaCl	
158	Murine Norovirus	Polymerase bound to NF023	3UPF	2.60	C2	Hanging drop (20°C)	1.5 M $(NH_4)_2SO_4$,<br>11% Glycerol<br>50 mM Tris pH 8.4	
159	Murine Norovirus	Polymerase with Suramin	3UR0	2.45	C2	Hanging drop (20°C)	1.3 M $(NH_4)_2SO_4$<br>10% Glycerol<br>0.1 M Tris, pH 8.4	
160	Murine Norovirus	Polymerase with a suramin derivative	4NRU	2.30	$P 2_1$	Microbatch	1.6 M $(NH_4)_2SO_4$<br>12% Glycerol<br>0.1 M Tris-HCl pH 8.4	(Croci, Pezzullo, *et al.*, 2014)
161	Murine Norovirus	RdRp in complex with PPNDS	4O4R	2.40	C2	Macrobatch (20°C)	1.6 M $(NH_4)_2SO_4$<br>12% Glycerol<br>0.1 M Tris-HCl pH 8.4	(Croci, Tarantino, *et al.*, 2014)
162	Poliovirus	Polymerase full length apo	1RA6	2.00	$P6_5$	Hanging drop (16°C)	1.5 M Na Acetate, 0.1 M Cacodylic acid, pH 7.1	(Thompson & Peersen, 2004)
163	Poliovirus	Polymerase with GTP	1RA7	2.35	$P6_5$	Hanging drop (16°C)	2 mM DTT	

Biology Open • Supplementary information

164	Poliovirus	Pol with 68 residues N-term truncation	1RAJ	2.50	$P3_12_1$	Hanging drop (16°C)	1.5 M Ammonium Formate, 0.1 M NaCl, 0.05 M HEPES, pH 7.0, 2 mM DTT	
165	Poliovirus	3D polymerase	1RDR	2.40	$P3_12_1$	Hanging drop (16°C)		
166	Poliovirus	Polymerase G1A mutant	1TQL	2.30	$P6_5$	Hanging drop (16°C)	2M Na-Acetate, 2 mM DTT<br>0.1 M Cacodylic acid, pH 7.1	
167	Poliovirus	RdRp 3Dpol G64S mutant	2IJF	3.00	$P6_5$	Hanging drop (20°C)	2 M NaOAc<br>0.1 M HEPES, pH 7.0	(Marcotte *et al.*, 2007)
168	Poliovirus	Polymerase with ATP and Mg^{2+}	2ILY	2.60	$P6_5$	Hanging drop (16°C)	2 M Na Acetate<br>0.1 M Cacodylic acid, pH 7.1<br>2 mM DTT	(Thompson *et al.*, 2007)
169	Poliovirus	Polymerase with GTP and Mn^{2+}	2ILZ	2.50	$P6_5$	Hanging drop (16°C)		
170	Poliovirus	Polymerase with CTP and Mn^{2+}	2IM0	2.25	$P6_5$	Hanging drop (16°C)		
171	Poliovirus	Polymerase with CTP and Mn^{2+}	2IM1	2.50	$P6_5$	Hanging drop (16°C)		

Biology Open • Supplementary information

172	Poliovirus	Polymerase with UTP and Mn^{2+}	2IM2	2.35	$P6_5$	Hanging drop (16°C)		
173	Poliovirus	Polymerase with UTP and Mn^{2+}	2IM3	2.60	$P6_5$	Hanging drop (16°C)		
174	Poliovirus	Polymerase C290I loop mutant	4NLO	2.20	$P6_5$	Hanging drop (16°C)	0.25 M Na Acetate<br>0.1 M Cacodylic acid, pH 7.0<br>2 mM DTT, 30% PEG 400	(Sholders & Peersen, 2014)
175	Poliovirus	Polymerase C290V loop mutant	4NLP	2.20	$P6_5$	Hanging drop (16°C)		
176	Poliovirus	Polymerase C290F loop mutant	4NLQ	2.30	$P6_5$	Hanging drop (16°C)		
177	Poliovirus	Polymerase C290S loop mutant	4NLR	1.85	$P6_5$	Hanging drop (16°C)		
178	Poliovirus	Polymerase S288A loop mutant	4NLS	2.00	$P6_5$	Hanging drop (16°C)		
179	Poliovirus	Polymerase S291P loop	4NLT	2.50	$P6_5$	Hanging drop (16°C)		

Biology Open • Supplementary information

		mutant						
180	Poliovirus	Polymerase G289A loop mutant	4NLU	1.96	$P6_5$	Hanging drop (16°C)		
181	Poliovirus	Polymerase G289A/C290F loop mutant	4NLV	2.30	$P6_5$	Hanging drop (16°C)		
182	Poliovirus	Polymerase G289A/C290I loop mutant	4NLW	2.10	$P6_5$	Hanging drop (16°C)		
183	Poliovirus	Polymerase G289A/C290 V loop mutant	4NLX	2.60	$P6_5$	Hanging drop (16°C)		
184	Poliovirus	Polymerase C290E loop mutant	4NLY	2.30	$P6_5$	Hanging drop (16°C)		
185	Poliovirus	RdRp low-fidelity mutant 3D pol H273R	4R0E	3.00	$P6_5$	(20°C)	2 M Na Acetate<br>0.1 M Na Cacodylate, pH 6.8	(Moustafa *et al.*, 2014)
186	Pseudomonas phage Phi6	RdRp	1HHS	2.00	$P2_1$		10% PEG 8000<br>0.1 M MES<br>2 mM $MnCl_2$	(Butcher *et al.*, 2001)

Biology Open • Supplementary information

187	Pseudomonas phage Phi6	RdRp	2JLF	3.20	P32		15-20% PEG 4000<br>8.5% Isopropanol<br>15% Glycerol<br>0.1 M HEPES, pH 7.5	(Poranen *et al.*, 2008)
188	Pseudomonas phage Phi6	RdRp	4A8F	3.30	$P2_1$		20% PEG 4000<br>0.1 M HEPES, pH 7.5<br>8.5% Isopropanol<br>15% Glycerol<br>2 mM $MnCl_2$	(Wright *et al.*, 2012)
189	Pseudomonas phage Phi6	RdRp	4A8K	2.90	$P2_1$			
190	Pseudomonas phage Phi6	RdRp	4A8M	2.90	$P2_1$			
191	Pseudomonas phage Phi6	RdRp	4A8O	2.67	$P2_1$			
192	Pseudomonas phage Phi6	RdRp	4A8Q	3.00	$P2_1$		20% PEG 2000<br>0.1 M MES, pH 6.5	
193	Pseudomonas phage Phi6	RdRp	4A8S	2.90	$P2_1$			
194	Pseudomonas phage Phi6	RdRp	4A8Y	3.40	$P2_1$			
195	Rabbit Hemorrhagic	RdRp with Lu^{3+}	1KHV	2.50	$P2_12_12_1$	Hanging drop (25°C)	10–12% PEG 8000<br>0.1 M Tris-HCl, pH 7.5	(Ng *et al.*, 2002)

Biology Open • Supplementary information

	Disease Virus						0.2 M Na thiocyanate<br>0.1 M L-proline<br>15% Glycerol<br>7% 1,6-hexanediol,<br>0.1% CHAPS,<br>5 mM $CaCl_2$<br>2 mM $MgCl_2$	
196	Rabbit Hemorrhagic Disease Virus	RdRp with Mn^{2+}	1KHW	2.70	$P2_12_12_1$	Hanging drop (25°C)	10-12% PEG 8000<br>0.1 M Tris, pH 7.5<br>0.2 M sodium thiocyanate<br>0.1 M L-proline<br>15% (w/v) glycerol<br>7% (v/v) 1,6-hexanediol<br>0.1% (w/v) CHAPS<br>5 mM $CaCl_2$,<br>2 mM $MgCl_2$	
197	Thielavia terres	RdRp QDE-1	5FSW	3.19	$P2_1$		0.1 M Tris, pH 8.0<br>75 mM NaCl<br>10% PEG 10000	(Qian *et al.*, 2016)

Biology Open • Supplementary information

198	Thosea asigna virus	RdRp complexed with CDP	5CX6	2.10	$P2_12_12_1$	Sitting drop (20°C)	12% PEG 8000<br>0.75 M Li_2SO_4	(Ferrero *et al.*, 2015)
199	Thosea asigna virus	RdRp with ATP and ssRNA	5CYR	3.50	I222	Sitting drop (20°C)		
200	Thosea asigna virus	RdRp complexed with Lu^{3+}	4XHA	3.00	$C222_1$	Sitting drop (20°C)		
201	Thosea asigna virus	RdRp native	4XHI	2.15	$P2_12_12$	Sitting drop (20°C)		

Biology Open • Supplementary information

Table S2 The calculated volume for each stock solution used in the distinct conditions of the screen is provided (Total volume: 10 ml).

	1	2	3	4	5	6	7	8	9	10	11	12
**A**	0.5 ml MES, pH 5.0<br>7.3 ml NaCl<br>1.5 ml Glycerol<br>0.7 ml H_2O	0.5 ml MMT, pH 5.5<br>7.3 ml NaCl<br>1.5 ml Glycerol<br>0.7 ml H_2O	0.5 ml $(CH_3)_2AsO_2Na$, pH 6.5<br>7.3 ml NaCl<br>1.5 ml Glycerol<br>0.7 ml H_2O	0.85 ml HEPES, pH 7.0<br>7.3 ml NaCl<br>1.5 ml Glycerol<br>0.35 ml H_2O	0.85 ml Tris, pH 7.5<br>3.18 ml $(NH_4)_2SO_4$<br>2.5 ml Glycerol<br>3.46 ml H_2O	0.5 ml Tris, pH 7.5<br>3.22 ml $(NH_4)_2SO_4$<br>2.45 ml Glycerol<br>3.83 ml H_2O	0.5 ml Tris, pH 8.0<br>3.22 ml $(NH_4)_2SO_4$<br>2.45 ml Glycerol<br>3.83 ml H_2O	0.5 ml Tris, pH 8.5<br>3.22 ml $(NH_4)_2SO_4$<br>2.45 ml Glycerol<br>3.83 ml H_2O	0.5 ml SPG, pH 9.0<br>3.22 ml $(NH_4)_2SO_4$<br>2.45 ml Glycerol<br>3.83 ml H_2O	1 ml MES, pH 5.0<br>0.02 ml $MnCl_2$<br>0.02 ml $MgCl_2$<br>3.25 ml $(NH_4)_2SO_4$<br>5.71 ml H_2O	1 ml Bis-Tris, pH 6.1<br>0.01 ml DTT<br>0.2 ml $NiSO_4$<br>30.25 ml $(NH_4)_2SO_4$<br>5.54 ml H_2O	1 ml $(CH_3)_2AsO_2Na$, pH 6.5<br>2.5 ml $(NH_4)_2SO_4$<br>6.5 ml H_2O
**B**	1 ml $(CH_3)_2AsO_2Na$, pH 6.5<br>0.4 ml NaCl<br>5 ml $(NH_4)_2SO_4$<br>3.6 ml H_2O	0.5 ml HEPES, pH 7.0<br>0.2 ml NaCl<br>3.75 ml $(NH_4)_2SO_4$<br>0.02 ml DTT<br>5.53 ml H_2O	0.5 ml HEPES, pH 7.5<br>0.2 ml NaCl<br>3.75 ml $(NH_4)_2SO_4$<br>0.02 ml DTT<br>5.53 ml H_2O	0.5 ml Tris, pH 8.0<br>3.75 ml $(NH_4)_2SO_4$<br>10.1 ml Glycerol<br>4.65 ml H_2O	0.5 ml Tris, pH 8.5<br>3.75 ml $(NH_4)_2SO_4$<br>10.1 ml Glycerol<br>4.65 ml H_2O	2 ml CHES, pH 9.5<br>0.4 ml NaCl<br>30.15 ml $(NH_4)_2SO_4$<br>4.45 ml H_2O	2 ml CAPS, pH 10.0<br>1 ml Li2SO4<br>5ml $(NH_4)_2SO_4$<br>2 ml H_2O	1 ml MMT, pH 5.0<br>5.333 ml K/Na Tartrate<br>3.667 ml H_2O	1 ml MMT, pH 5.5<br>5.333 ml K/Na Tartrate<br>0.05 ml of PEG 550 MME<br>3.617 ml H_2O	1 ml MMT, pH 6<br>5.333 ml K/Na Tartrate<br>3.667 ml H_2O	1 ml $(CH_3)_2AsO_2Na$, pH 6.5<br>5.333 ml K/Na Tartrate<br>3.667 ml H_2O	1 ml HEPES, pH 7.0<br>5.333 ml K/Na Tartrate<br>0.1 ml of PEG 400<br>3.567 ml H_2O
**C**	1 ml HEPES, pH 7.5<br>5.333 ml K/Na Tartrate<br>3.667 ml H_2O	1 ml Tris-HCl, pH 8.5<br>5.333 ml K/Na Tartrate<br>0.1 ml PEG 5000 MME<br>3.567 ml H_2O	1 ml Tris-HCl, pH 8.5<br>5.333 ml K/Na Tartrate<br>0.2 ml PEG 2000 MME<br>3.467 ml H_2O	1 ml $(CH_3)_2AsO_2Na$, pH 6.5<br>0.25 ml NaCl<br>4 ml $Na_3C_6H_5O_7$<br>4.75 ml H_2O	1 ml HEPES, pH 7.5<br>0.1 ml NaI<br>3.823 ml $C_3H_2O_4Na_2$<br>5.077 ml H_2O	1 ml HEPES, pH 7.0<br>6.67 ml $C_2H_3NaO_2$<br>0.1 ml PEG 550 MME<br>20.233 ml H_2O	1 ml $(CH_3)_2AsO_2Na$, pH 7.1<br>6.67 ml $C_2H_3NaO_2$<br>2.33 ml H_2O	0.5 ml MES, pH 5.0<br>2 ml NaCl<br>2 ml PEG 400<br>0.05 ml DTT<br>5.45 ml H_2O	1 ml Bis-Tris propane, pH 6.5<br>00.125 ml $(NH_4)_2SO_4$<br>3 ml PEG 550 MME<br>5.875 ml H_2O	1 ml HEPES, pH 7.5<br>0.5 ml $MgCl_2$<br>3 ml PEG 550 MME<br>5.5 ml H_2O	1 ml HEPES, pH 7.5<br>0.5 ml $MnCl_2$<br>3 ml PEG 550 MME<br>5.5 ml H_2O	0.5 ml Tris, pH 7.5<br>0.2 ml NH_4OAc<br>2.5 ml PEG 550 MME<br>6.8 ml H_2O
**D**	1 ml Tris, pH 8.0<br>2.5 ml PEG 550 MME<br>6.5 ml H_2O	1 ml Tris, pH 8.0<br>2 ml PEG 550 MME<br>7 ml H_2O	1 ml Tris, pH 8.5<br>2 ml PEG 550 MME<br>7 ml H_2O	1 ml Bicine, pH 9.0<br>2.5 ml PEG 550 MME<br>6.5 ml H_2O	0.5 ml MES, pH 6.5<br>0.5 ml $(NH_4)_2SO_4$<br>0.25 ml NH_4OAc<br>2.5 ml PEG 1000<br>60.25 ml dH_2O	1 ml MES, pH 8.0<br>2.5 ml PEG 1500<br>6.5 ml H_2O	1 ml MES, pH 6.5<br>5 ml PEG 2000<br>4 ml H_2O	00.1 ml NH_4OAc, pH 5.0<br>0.5 ml $(NH_4)_2SO_4$<br>6 ml PEG 2000 MME<br>3.4 ml dH_2O	1 ml HEPES, pH 7.0<br>8.333 ml $C_4H_6O_4$<br>00.1 ml PEG 2000 MME<br>0.567 ml H_2O	1 ml $Na_3C_6H_5O_7$, pH 5.5<br>3.6 ml PEG 3350<br>1.4 ml Glycerol<br>4 ml H_2O	1 ml Bis-Tris propane, pH 6.0<br>0.5 ml NH_4OAc<br>6 ml PEG 3350<br>2.5 ml H_2O	1 ml MES, pH 6.0<br>4 ml $LiNO_3$<br>2 ml PEG 3350<br>3 ml H_2O
**E**	1 ml Bis-Tris Propane, pH 7.5<br>2 ml $Na_3C_6H_5O_7$<br>4 ml PEG 3350<br>3 ml H_2O	1 ml Bis-Tris Propane, pH 7.5<br>1 ml $Na_3C_6H_5O_7$<br>4 ml PEG 3350<br>0.5 ml MPD<br>0.05 ml $MnCl_2$<br>0.033 ml ATP<br>0.02 ml NaOH<br>3.397 ml H_2O	1 ml MMT Buffer pH 8.0<br>3.5 ml $LiNO_3$<br>2.4 ml PEG 3350<br>30.1 ml H_2O	0.333 ml $C_2H_3NaO_2$, pH 4.7<br>0.125 ml $(NH_4)_2SO_4$<br>4 ml PEG 4000<br>0.05 ml DTT<br>5.492 ml H_2O	0.5 ml $Na_3C_6H_5O_7$, pH 4.9<br>50.2 ml PEG 4000<br>0.75 ml Glycerol<br>3.550 ml H_2O	0.5 ml MES, pH 5.0<br>0.05 ml DTT<br>4 ml PEG 4000<br>1 ml Glycerol<br>4.45 ml H_2O	0.5 ml MES, pH 5.0<br>4 ml PEG 4000, 0.5 ml Glycerol<br>5 ml H_2O	1 ml $Na_3C_6H_5O_7$, pH 5.0<br>4 ml PEG 4000<br>0.05 ml DTT<br>1 ml Glycerol<br>3.950 ml H_2O	0.333 ml $C_2H_3NaO_2$, pH 5.0<br>0.6 ml NaCl<br>3.6 ml PEG 4000<br>0.004 ml β-mercaptoethanol<br>5.463 ml H_2O	0.333 ml $C_2H_3NaO_2$, pH 5.0<br>0.6 ml NaCl<br>30.2 ml PEG 4000<br>1 ml Glycerol<br>0.004 ml β-mercaptoethanol<br>4.863 ml H_2O	0.5 ml $Na_3C_6H_5O_7$, pH 5.6<br>0.2 ml NH_4OAc<br>6.6 ml PEG 4000<br>0.4 ml γ-butyrolactone<br>2.3 ml H_2O	1 ml $Na_3C_6H_5O_7$, pH 5.6<br>0.087 ml $(NH_4)_2SO_4$<br>8 ml PEG 4000<br>0.5 ml Glycerol<br>0.05 ml DTT<br>0.363 ml H_2O
**F**	1 ml MES, pH 6.0<br>2 ml Mg(CH3COO)2<br>6 ml PEG 4000 0.4 ml γ- butyrolactone<br>0.6 ml H_2O	0.333 ml $C_2H_3NaO_2$, pH 6.0<br>0.6 ml NaCl<br>2.8 ml PEG 4000<br>0.004 ml β-mercaptoehanol<br>60.263 ml dH_2O	0.5 ml $Na_3C_6H_5O_7$, pH 6.5<br>3.4 ml PEG 4000<br>60.1 ml H_2O	0.333 ml $C_2H_3NaO_2$, pH 6.5<br>0.350 ml NH_4OAc<br>5 ml PEG 4000<br>4.317 ml H_2O	1 ml $(CH_3)_2AsO_2Na$, pH 6.5<br>2 ml $Mg(CH_3COO)_2$<br>6 ml PEG 4000<br>1 ml H_2O	0.5 ml $Na_3C_6H_5O_7$, pH 6.8<br>0.8 ml PEG 4000<br>0.7 ml Isopropanol<br>8 ml H_2O	0.5 ml Tris, pH 7.5<br>50.2 ml PEG 4000<br>0.75 ml Glycerol<br>3.55 ml H_2O	1 ml Na-HEPES, pH 7.5<br>00.1 ml $CaCl_2$<br>0.4 ml NaCl<br>5 ml PEG 4000<br>1 ml Glycerol<br>2.5 ml H_2O	1 ml HEPES, pH 7.5<br>4 ml PEG 4000<br>0.85 ml Isopropanol<br>1.5 ml Glycerol<br>2.65 ml H_2O	1 ml HEPES, pH 7.5<br>0.02 ml $MnCl_2$<br>4 ml PEG 4000<br>0.85 ml Isopropanol<br>1.5 ml Glycerol<br>2.63 ml H_2O	1 ml Tris, pH 8.0<br>0.02 ml $MnCl_2$<br>4 ml PEG 4000<br>0.85 ml Isopropanol<br>1.5 ml Glycerol<br>2.63 ml H_2O	1 ml Tris, pH 8.0<br>0.02 ml $MnCl_2$<br>4 ml PEG 4000<br>0.85 ml Isopropanol<br>1.5 ml Glycerol<br>2.63 ml H_2O
**G**	1 ml Bicine, pH 9.0<br>0.02 ml $MnCl_2$<br>4 ml PEG 4000<br>0.85 ml Isopropanol<br>1.5 ml Glycerol<br>2.63 ml H_2O	3.75 ml Li_2SO_4<br>2.4 ml PEG 8000<br>3.85 ml H_2O	0.333 ml $C_2H_3NaO_2$, pH 5.0<br>0.5 ml $(NH_4)_2SO_4$<br>5.4 ml PEG 5000 MME<br>3.767 ml H_2O	1 ml MES, pH 5<br>1 ml $(NH_4)_2SO_4$<br>40.2 ml PEG 5000 MME<br>1 ml Glycerol<br>2.8 ml dH_2O	0.5 ml $Na_3C_6H_5O_7$, pH 5.5<br>1.6 ml PEG 8000<br>0.5 ml Isopropanol<br>7.4 ml H_2O	1 ml MES, pH 6.0<br>00.1 ml $MnCl_2$<br>2.8 ml PEG 8000<br>1.4 ml Isopropanol<br>0.05 ml TCEP<br>4.65 ml H_2O	1 ml $(CH_3)_2AsO_2Na$, pH 6.4<br>2 ml $Mg(CH_3COO)_2$<br>3 ml PEG 8000<br>4 ml H_2O	1 ml MES, pH 6.5<br>0.5 ml $(NH_4)_2SO_4$<br>5 ml PEG 5000 MME<br>3.5 ml H_2O	1 ml M $Na_3C_6H_5O_7$, pH 6.5<br>1 ml PEG 8000, 0.5 ml Isopropanol<br>7.5 ml H_2O	1 ml Buffer System 1, pH 6.5 (Morpheus)<br>0.2 ml $C_4H_6O_5$<br>2 ml PEG 8000<br>2 ml Ethylene Glycol<br>4.8 ml H_2O	1 ml MES, pH 6.5<br>0.02 ml M $MnCl_2$<br>2 ml PEG 8000<br>6.98 ml H_2O	1 ml HEPES pH 7.5<br>2 ml PEG 8000<br>0.8 ml Ethylene Glycol<br>60.2 ml H_2O
**H**	1 ml Tris, pH 7.5<br>0.01 ml Tween-20<br>0.05 ml $CaCl_2$<br>0.02 ml $MgCl_2$<br>2.4 ml PEG 8000<br>1.5 ml Glycerol<br>1.4 ml 1,6-hexanediol	0.5 ml Tris, pH 7.5<br>0.5 ml $(NH_4)_2SO_4$<br>4.8 ml PEG 8000<br>1.5 ml Glycerol<br>2.7 mldH_2O	0.5 ml Tris, pH 7.5<br>0.25 ml $(NH_4)_2SO_4$<br>0.2 ml $MgSO_4$<br>4.8 ml PEG 8000<br>1.5 ml Glycerol<br>2.75 ml H_2O	0.5 ml Tris, pH 8.0<br>0.5 ml $(NH_4)_2SO_4$<br>4.8 ml PEG 8000<br>1.5 ml Glycerol<br>2.7 ml H_2O	0.5 ml Tris, pH 8.5<br>0.5 ml $(NH_4)_2SO_4$<br>4.8 ml PEG 8000<br>1.5 ml Glycerol<br>2.7 ml H_2O	1 ml Tris, pH 7.5<br>0.15 ml NaCl<br>2 ml PEG 10000<br>6.85 ml H_2O	1 ml Tris, pH 8.0<br>0.15 ml NaCl<br>2 ml PEG 10000<br>6.85 ml H_2O	1 ml Tris, pH 8.5<br>0.15 ml NaCl<br>2 ml PEG 10000<br>6.85 ml H_2O	2 ml NaH_2PO_4, pH 7.0<br>1.4 ml PEG 20000<br>6.6 ml H_2O	2 ml HEPES, pH 7.5<br>1.4 ml PEG 20000<br>6.6 ml H_2O	2 ml Tris, pH 8.0<br>1.4 ml PEG 20000<br>6.6 ml H_2O	2 ml HEPES, pH 8.5<br>1.4 ml PEG 20000<br>6.6 ml H_2O

Table S3 List of chemicals used in the preparation of the crystallization screen. The table provides the names, source of the chemicals and their catalogue numbers and stock solutions.

Reagent	Company	Catalogue No.	Stock solution
1,6-Hexanediol	Sigma	240117	50% (v/v)
Ammonium Acetate	Acros Organics /Fisher	AC401152500	1 M
Ammonium Sulfate	Sigma	A4418	4 M
ATP	Acros Organics /Fisher	AC102800100	6 M
Bicine	Bio Basic Inc.	BB0266	1 M
Bis-Tris Propane	Melford	B7510	1 M
Buffer System 1 - Morpheus	Molecular Dimension	MD2-100-100	100% (v/v)
γ-Butyrolactone	Aldrich	B103608	100% (w/v)
Calcium Chloride	Sigma-Aldrich	C3306	1 M
CHAPS	Melford	B2006	0.5 M
CHES	Bio Basic Inc.	CB0115	0.5 M
DTT	Fluka	43817	1 M
Ethylene Glycol	Alfa Aesar	A11591	100% (w/v)
Glycerol	Melford	G1345	100% (w/v)
HEPES	Melford	B2001	1 M
Isopropanol	Fisher Scientific	BP2618	100% (v/v)
Lithium Nitrate	Sigma-Aldrich	227986	1 M
Lithium Sulfate	Acros Organics /Fisher	218331000	2 M
L-Proline	Melford	P0717	100 mM
Magnesium Acetate	Sigma-Aldrich	2286448	1 M
Magnesium Chloride	Melford	M0533	1 M
Malic Acid	Acros Organics /Fisher	125252500	1 M
Manganese Chloride	Alfa Aesar	11868	1 M
MES	Sigma-Aldrich	M2933	1 M
MPD	Sigma-Aldrich	112100	100% (w/v)
Nickel Sulfate	Fisher Scientific	10568810	1 M

PEG 400	Sigma-Aldrich	202398	100% (w/v)
PEG 1000	Acros Organics /Fisher	AC192250010	50% (w/v)
PEG 1500	Fluka	81210	50% (w/v)
PEG 2000	VWR	200007-386	50% (w/v)
PEG 3350	Sigma-Aldrich	202444	50% (w/v)
PEG 4000	Sigma-Aldrich	95904	50% (w/v)
PEG 8000	Sigma-Aldrich	89510	50% (w/v)
PEG 10000	Sigma-Aldrich	81280	50% (w/v)
PEG 20000	Sigma-Aldrich	95172	30% (w/v)
PEG 550 MME	Aldrich	202487	100% (w/v)
PEG 2000 MME	VWR	AAA17925-30	30% (w/v)
PEG 5000 MME	Sigma-Aldrich	81323	50% (w/v)
Sodium Acetate	Sigma-Aldrich	52889	3 M
Sodium Cacodylate	Sigma-Aldrich	C4945	1 M
Sodium Chloride	Sigma-Aldrich	S3014	5 M
Sodium Citrate	Alfa Aesar	L12557	1 M
Sodium Dihydrogen Phosphate	Melford	S2318	1 M
Sodium Hydroxide	Sigma-Aldrich	221465	5 M
Sodium Iodide	Millipore Merck	106523	2 M
Sodium Malonate	Sigma-Aldrich	63409	3.4 M
Sodium Potassium Tartrate	Sigma-Aldrich	217255	2 M
Succinic Acid	Acros Organics /Fisher	AC158742500	2 M
TCEP	Melford	T2650	1 M
Tris	Melford	B2005	1 M
Tri-Sodium Citrate	Acros Organics /Fisher	227130010	1 M
Tween-20	Millipore Merck	655204	100% (w/v)
β-Mercaptoethanol	Acros Organics /Fisher	125472500	14.3 M

Table S4 Summary of approximate diffraction limits obtained for Dengue RdRp3 crystals in various well conditions of the crystallization screen.

Well Number	*Resolution*
A3	No diffraction
A4	No diffraction
A6	10.0
A11	10.0
A12	8.0
B4	3.0
B5	15.0
B6	No diffraction
B9	2.3
B11	15.0
B12	3.1
C2	2.3
C3	2.7
C6	2.2
D1	2.1
D2	2.0
D3	15.0
D5	2.7

Well Number	*Resolution*
D6	10.0
D7	3.0
D11	12.0
E1	7.0
E2	4.5
E4	2.9
E6	20.0
E8	4.0
E12	5.0
F1	15.0
F2	12.0
F3	17.0
F9	14.0
G1	2.3
G2	2.3
G4	6.0
G8	8.0
H4	No diffraction

Biology Open • Supplementary information

Table S5 Data collection and structure refinement statistics of dengue RdRp3 structures obtained from different crystallization conditions in the RdRp Screen.

Well Number	**B4**	**B9**	**B12**	**C2**	**C3**	**C6**	**D1**	**D2**	**D5**	**D7**	**E6**	**G1**	**G2**
Unit-cell parameters	120.6, 57.8, 120.8, 90, 96.4, 90	162.9, 180.8, 58.1, 90, 90, 90	162.1, 180.0, 57.9, 90, 90, 90	160.8, 177.9, 57.9, 90, 90, 90	162.5, 179.6, 58.1, 90, 90, 90	161.4, 178.3, 58.0, 90, 90, 90	162.4, 180.0, 58.3, 90, 90, 90	161.5, 179.0, 58.0, 90, 90, 90	163.5, 180.6, 58.3, 90, 90, 90	163.8, 181.0, 58.3, 90, 90, 90	162.7, 179.7, 58.1, 90, 90, 90	162.8, 179.4, 58.1, 90, 90, 90	162.1, 179.5, 58.1, 90, 90, 90
Space group	$P2_1$	$C222_1$	$C222_1$	$C222_1$	$C222_1$	$C222_1$	$C222_1$	$C222_1$	$C222_1$	$C222_1$	$C222_1$	$C222_1$	$C222_1$
Resolution range	48.6 – 3.0	60.5 – 2.3	48.7 – 3.0	80.4 – 2.3	48.8 – 2.7	48.6 – 2.2	60.3 – 2.1	48.7 – 2.0	49.0 – 2.7	81.9 – 3.0	60.3 – 2.9	48.8 – 2.3	81.1 – 2.3
Total reflections	100931 (14551)	146729 (22230)	72867 (9527)	166100 (25269)	86350 (13120)	242356 (33763)	167787 (23649)	248250 (37775)	148497 (21746)	58720 (7835)	76136 (9760)	168413 (25106)	690005 (7092)
Unique reflections	33463 (4841)	38149 (5508)	15767 (2268)	37243 (5371)	23467 (3404)	42774 (6205)	49636 (7190)	55928 (8180)	25451 (3633)	17272 (2491)	19297 (2775)	37242 (5486)	36741 (3712)
Completeness (%)	99.4 (99.7)	98.9 (99.1)	99.6 (99.6)	99.7 (99.9)	98.8 (99.7)	99.5 (99.7)	99.0 (99.6)	98.1 (99.6)	99.5 (99.6)	97.3 (98.0)	99.7 (99.7)	99.2 (97.6)	96.2 (98.6)
Multiplicity	3.0 (3.0)	3.8 (4.0)	4.6 (4.2)	4.5 (4.7)	3.7 (3.8)	5.7 (5.4)	3.4 (3.3)	4.4 (4.6)	5.8 (6.0)	3.4 (3.1)	3.9 (3.5)	4.5 (4.6)	11.0 (3.0)
R merge (%)	13.9 (44.0)	8.6 (50.4)	15.5 (58.8)	10.0 (38.8)	13.6 (50.4)	7.7 (70.2)	8.4 (42.0)	9.8 (72.0)	10.2 (60.3)	14.4 (58.5)	10.8 (15.8)	6.6 (50.1)	4.7 (26.9)
I/∑I	6.8 (2.4)	10.9 (3.2)	8.2 (2.0)	9.3 (3.1)	10.6 (4.3)	15.1 (2.4)	8.5 (2.4)	9.8 (2.0)	14.4 (3.3)	6.4 (1.9)	9.4 (5.2)	13.9 (2.8)	14.5 (4.5)
R work/R free (%)	17.5/25.2	18.4/24.4	18.3/26.1	20.8/26.2	17.4/24.3	20.0/23.4	20.2/26.4	24.6/27.0	17.7/22.9	18.6/26.5	21.6/28.4	17.7/22.5	17.6/22.4
Wilson B (^{2})	54.1	33.0	58.4	28.1	27.8	28.7	25.9	24.3	28.6	52.6	33.2	41.0	33.3
r.m.s.d. bond length	0.018	0.013	0.016	0.013	0.013	0.012	0.012	0.013	0.013	0.017	0.014	0.012	0.012
r.m.s.d. bond angles	1.706	1.213	1.704	1.298	1.295	1.140	1.152	1.331	1.215	1.742	1.588	1.177	1.240
Ramachandran													
Favoured	90.0	96.0	97.0	95.3	94.7	96.2	96.7	96.7	96.7	88.1	96.6	95.7	96.3
Allowed	8.4	3.2	1.8	4.6	4.6	33	2.9	2.5	2.6	10.2	2.50	4.1	3.3
Outliers	1.6	0.9	1.2	0.2	0.7	0.5	0.4	1.3	0.7	1.8	0.9	0.2	0.4

Table S6 Details of missing regions in the structures, PEG and water molecules in the structure of dengue RdRp3 solved from the screen. Interestingly, the flexible regions in motif G (~ 454 – 469) and motif F (~ 406 – 419) are missing in all the structures consistently proving all the structures are in the closed confirmation.

Well Number on plate	**B4**	**B9**	**B12**	**C2**	**C3**	**C6**	**D1**	**D2**	**D5**	**D7**	**E4**	**G1**	**G2**
Resolution (Å)	3.0	2.3	3.0	2.3	2.7	2.2	2.1	2.0	2.7	3.0	2.9	2.3	2.3
Missing regions	Chain A<br>311 – 317<br>344 – 354<br>406 – 418<br>451 – 469<br>583 – 586<br><br>Chain B<br>310 – 317<br>344 – 356<br>406 – 418<br>453 – 469<br>583 –588	311 – 317<br>406 – 417<br>454 – 469	312 – 315<br>406 – 419<br>454 – 469	310 – 318<br>343 – 353<br>407 – 419<br>454 – 469<br>581 – 588	311 – 317<br>343 – 353<br>407 – 419<br>454 – 469<br>581 – 588	311 – 317<br>344 – 349<br>406 – 419<br>454 – 469<br>581 – 586	311 – 318<br>343 – 353<br>406 – 419<br>454 – 473<br>581 – 588	311 – 318<br>344 – 348<br>407 – 419<br>454 – 469<br>583 – 585	406 – 419<br>454 – 469	311 – 317<br>406 – 418<br>454 – 469	311 – 317<br>406 – 418<br>455 – 469	314 – 316<br>406 – 416<br>456 – 469	312 – 318<br>405 – 418<br>454 – 469
No. of PEG molecules	none	3	6	5	5	3	2	2	3<br>(1 –P6G)	5	4	5	6
No. of water molecules	35	348	90	222	223	334	871	367	256	92	213	254	272

Biology Open • Supplementary information

References

Ago, H., Adachi, T., Yoshida, A., Yamamoto, M., Habuka, N., Yatsunami, K. & Miyano, M. (1999). *Structure* **7**, 1417-1426.

Alam, I., Lee, J. H., Cho, K. J., Han, K. R., Yang, J. M., Chung, M. S. & Kim, K. H. (2012). *Virology* **426**, 143-151.

Ando, I., Adachi, T., Ogura, N., Toyonaga, Y., Sugimoto, K., Abe, H., Kamada, M. & Noguchi, T. (2012). *Antimicrob Agents Chemother* **56**, 4250-4256.

Antonysamy, S. S., Aubol, B., Blaney, J., Browner, M. F., Giannetti, A. M., Harris, S. F., Hebert, N., Hendle, J., Hopkins, S., Jefferson, E., Kissinger, C., Leveque, V., Marciano, D., McGee, E., Najera, I., Nolan, B., Tomimoto, M., Torres, E. & Wright, T. (2008). *Bioorg Med Chem Lett* **18**, 2990-2995.

Appleby, T. C., Luecke, H., Shim, J. H., Wu, J. Z., Cheney, I. W., Zhong, W., Vogeley, L., Hong, Z. & Yao, N. (2005). *J Virol* **79**, 277-288.

Appleby, T. C., Perry, J. K., Murakami, E., Barauskas, O., Feng, J., Cho, A., Fox, D., 3rd, Wetmore, D. R., McGrath, M. E., Ray, A. S., Sofia, M. J., Swaminathan, S. & Edwards, T. E. (2015). *Science* **347**, 771-775.

Bahar, M. W., Sarin, L. P., Graham, S. C., Pang, J., Bamford, D. H., Stuart, D. I. & Grimes, J. M. (2013). *J Virol* **87**, 3229-3236.

Bressanelli, S., Tomei, L., Rey, F. A. & De Francesco, R. (2002). *J Virol* **76**, 3482-3492.

Butcher, S. J., Grimes, J. M., Makeyev, E. V., Bamford, D. H. & Stuart, D. I. (2001). *Nature* **410**, 235-240.

Campagnola, G., McDonald, S., Beaucourt, S., Vignuzzi, M. & Peersen, O. B. (2015). *J Virol* **89**, 275-286.

Canales, E., Carlson, J. S., Appleby, T., Fenaux, M., Lee, J., Tian, Y., Tirunagari, N., Wong, M. & Watkins, W. J. (2012). *Bioorg Med Chem Lett* **22**, 4288-4292.

Cherry, A. L., Dennis, C. A., Baron, A., Eisele, L. E., Thommes, P. A. & Jaeger, J. (2015). *J Virol* **89**, 2052-2063.

Choi, K. H., Gallei, A., Becher, P. & Rossmann, M. G. (2006). *Structure* **14**, 1107-1113.

Croci, R., Pezzullo, M., Tarantino, D., Milani, M., Tsay, S. C., Sureshbabu, R., Tsai, Y. J., Mastrangelo, E., Rohayem, J., Bolognesi, M. & Hwu, J. R. (2014). *PLoS One* **9**, e91765.

Croci, R., Tarantino, D., Milani, M., Pezzullo, M., Rohayem, J., Bolognesi, M. & Mastrangelo, E. (2014). *FEBS Lett* **588**, 1720-1725.

de Vicente, J., Hendricks, R. T., Smith, D. B., Fell, J. B., Fischer, J., Spencer, S. R., Stengel, P. J., Mohr, P., Robinson, J. E., Blake, J. F., Hilgenkamp, R. K., Yee, C., Adjabeng, G., Elworthy, T. R., Li, J., Wang, B., Bamberg, J. T., Harris, S. F., Wong, A., Leveque, V. J., Najera, I., Le Pogam, S., Rajyaguru, S., Ao-Ieong, G., Alexandrova, L., Larrabee, S., Brandl, M., Briggs, A., Sukhtankar, S. & Farrell, R. (2009). *Bioorg Med Chem Lett* **19**, 5652-5656.

de Vicente, J., Hendricks, R. T., Smith, D. B., Fell, J. B., Fischer, J., Spencer, S. R., Stengel, P. J., Mohr, P., Robinson, J. E., Blake, J. F., Hilgenkamp, R. K., Yee, C., Adjabeng, G., Elworthy, T. R., Tracy, J., Chin, E., Li, J., Wang, B., Bamberg, J. T., Stephenson, R., Oshiro, C., Harris, S. F., Ghate, M., Leveque, V., Najera, I., Le Pogam, S., Rajyaguru, S., Ao-Ieong, G., Alexandrova, L., Larrabee, S., Brandl, M., Briggs, A., Sukhtankar, S., Farrell, R. & Xu, B. (2009). *Bioorg Med Chem Lett* **19**, 3642-3646.

de Vicente, J., Hendricks, R. T., Smith, D. B., Fell, J. B., Fischer, J., Spencer, S. R., Stengel, P. J., Mohr, P., Robinson, J. E., Blake, J. F., Hilgenkamp, R. K., Yee, C., Zhao, J., Elworthy, T. R., Tracy, J., Chin, E., Li, J., Lui, A., Wang, B., Oshiro, C., Harris, S. F., Ghate, M., Leveque, V. J., Najera, I., Le Pogam, S., Rajyaguru, S., Ao-Ieong, G., Alexandrova, L., Fitch, B., Brandl, M., Masjedizadeh, M., Wu, S. Y., de Keczer, S. & Voronin, T. (2009). *Bioorg Med Chem Lett* **19**, 5648-5651.

Di Marco, S., Volpari, C., Tomei, L., Altamura, S., Harper, S., Narjes, F., Koch, U., Rowley, M., De Francesco, R., Migliaccio, G. & Carfi, A. (2005). *J Biol Chem* **280**, 29765-29770.

Dragovich, P. S., Blazel, J. K., Ellis, D. A., Han, Q., Kamran, R., Kissinger, C. R., LeBrun, L. A., Li, L. S., Murphy, D. E., Noble, M., Patel, R. A., Ruebsam, F., Sergeeva, M. V., Shah, A. M.,

Showalter, R. E., Tran, C. V., Tsan, M., Webber, S. E., Kirkovsky, L. & Zhou, Y. (2008). *Bioorg Med Chem Lett* **18**, 5635-5639.
Ellis, D. A., Blazel, J. K., Tran, C. V., Ruebsam, F., Murphy, D. E., Li, L. S., Zhao, J., Zhou, Y., McGuire, H. M., Xiang, A. X., Webber, S. E., Zhao, Q., Han, Q., Kissinger, C. R., Lardy, M., Gobbi, A., Showalter, R. E., Shah, A. M., Tsan, M., Patel, R. A., LeBrun, L. A., Kamran, R., Bartkowski, D. M., Nolan, T. G., Norris, D. A., Sergeeva, M. V. & Kirkovsky, L. (2009). *Bioorg Med Chem Lett* **19**, 6047-6052.
Ellis, D. A., Blazel, J. K., Webber, S. E., Tran, C. V., Dragovich, P. S., Sun, Z., Ruebsam, F., McGuire, H. M., Xiang, A. X., Zhao, J., Li, L. S., Zhou, Y., Han, Q., Kissinger, C. R., Showalter, R. E., Lardy, M., Shah, A. M., Tsan, M., Patel, R., LeBrun, L. A., Kamran, R., Bartkowski, D. M., Nolan, T. G., Norris, D. A., Sergeeva, M. V. & Kirkovsky, L. (2008). *Bioorg Med Chem Lett* **18**, 4628-4632.
Ferrer-Orta, C., Arias, A., Agudo, R., Perez-Luque, R., Escarmis, C., Domingo, E. & Verdaguer, N. (2006). *EMBO J* **25**, 880-888.
Ferrer-Orta, C., Arias, A., Perez-Luque, R., Escarmis, C., Domingo, E. & Verdaguer, N. (2007). *Proc Natl Acad Sci U S A* **104**, 9463-9468.
Ferrer-Orta, C., de la Higuera, I., Caridi, F., Sanchez-Aparicio, M. T., Moreno, E., Perales, C., Singh, K., Sarafianos, S. G., Sobrino, F., Domingo, E. & Verdaguer, N. (2015). *J Virol* **89**, 6848-6859.
Ferrero, D. S., Buxaderas, M., Rodriguez, J. F. & Verdaguer, N. (2015). *PLoS Pathog* **11**, e1005265.
Garriga, D., Navarro, A., Querol-Audi, J., Abaitua, F., Rodriguez, J. F. & Verdaguer, N. (2007). *Proc Natl Acad Sci U S A* **104**, 20540-20545.
Graham, S. C., Sarin, L. P., Bahar, M. W., Myers, R. A., Stuart, D. I., Bamford, D. H. & Grimes, J. M. (2011). *PLoS Pathog* **7**, e1002085.
Gruez, A., Selisko, B., Roberts, M., Bricogne, G., Bussetta, C., Jabafi, I., Coutard, B., De Palma, A. M., Neyts, J. & Canard, B. (2008). *J Virol* **82**, 9577-9590.
Hang, J. Q., Yang, Y., Harris, S. F., Leveque, V., Whittington, H. J., Rajyaguru, S., Ao-Ieong, G., McCown, M. F., Wong, A., Giannetti, A. M., Le Pogam, S., Talamas, F., Cammack, N., Najera, I. & Klumpp, K. (2009). *J Biol Chem* **284**, 15517-15529.
Harrus, D., Ahmed-El-Sayed, N., Simister, P. C., Miller, S., Triconnet, M., Hagedorn, C. H., Mahias, K., Rey, F. A., Astier-Gin, T. & Bressanelli, S. (2010). *J Biol Chem* **285**, 32906-32918.
Hengrung, N., El Omari, K., Serna Martin, I., Vreede, F. T., Cusack, S., Rambo, R. P., Vonrhein, C., Bricogne, G., Stuart, D. I., Grimes, J. M. & Fodor, E. (2015). *Nature* **527**, 114-117.
Ikegashira, K., Oka, T., Hirashima, S., Noji, S., Yamanaka, H., Hara, Y., Adachi, T., Tsuruha, J., Doi, S., Hase, Y., Noguchi, T., Ando, I., Ogura, N., Ikeda, S. & Hashimoto, H. (2006). *J Med Chem* **49**, 6950-6953.
Kim, S. H., Tran, M. T., Ruebsam, F., Xiang, A. X., Ayida, B., McGuire, H., Ellis, D., Blazel, J., Tran, C. V., Murphy, D. E., Webber, S. E., Zhou, Y., Shah, A. M., Tsan, M., Showalter, R. E., Patel, R., Gobbi, A., LeBrun, L. A., Bartkowski, D. M., Nolan, T. G., Norris, D. A., Sergeeva, M. V., Kirkovsky, L., Zhao, Q., Han, Q. & Kissinger, C. R. (2008). *Bioorg Med Chem Lett* **18**, 4181-4185.
Kumar, D. V., Rai, R., Brameld, K. A., Somoza, J. R., Rajagopalan, R., Janc, J. W., Xia, Y. M., Ton, T. L., Shaghafi, M. B., Hu, H., Lehoux, I., To, N., Young, W. B. & Green, M. J. (2011). *Bioorg Med Chem Lett* **21**, 82-87.
Lam, A. M., Edwards, T. E., Mosley, R. T., Murakami, E., Bansal, S., Lugo, C., Bao, H., Otto, M. J., Sofia, M. J. & Furman, P. A. (2014). *Antimicrob Agents Chemother* **58**, 6861-6869.
LaPlante, S. R., Gillard, J. R., Jakalian, A., Aubry, N., Coulombe, R., Brochu, C., Tsantrizos, Y. S., Poirier, M., Kukolj, G. & Beaulieu, P. L. (2010). *J Am Chem Soc* **132**, 15204-15212.
Le Pogam, S., Kang, H., Harris, S. F., Leveque, V., Giannetti, A. M., Ali, S., Jiang, W. R., Rajyaguru, S., Tavares, G., Oshiro, C., Hendricks, T., Klumpp, K., Symons, J., Browner, M. F., Cammack, N. & Najera, I. (2006). *J Virol* **80**, 6146-6154.
Lee, J. H., Alam, I., Han, K. R., Cho, S., Shin, S., Kang, S., Yang, J. M. & Kim, K. H. (2011). *J Gen Virol* **92**, 1607-1616.
Lesburg, C. A., Cable, M. B., Ferrari, E., Hong, Z., Mannarino, A. F. & Weber, P. C. (1999). *Nat Struct Biol* **6**, 937-943.

Li, H., Tatlock, J., Linton, A., Gonzalez, J., Borchardt, A., Dragovich, P., Jewell, T., Prins, T., Zhou, R., Blazel, J., Parge, H., Love, R., Hickey, M., Doan, C., Shi, S., Duggal, R., Lewis, C. & Fuhrman, S. (2006). *Bioorg Med Chem Lett* **16**, 4834-4838.

Li, H., Tatlock, J., Linton, A., Gonzalez, J., Jewell, T., Patel, L., Ludlum, S., Drowns, M., Rahavendran, S. V., Skor, H., Hunter, R., Shi, S. T., Herlihy, K. J., Parge, H., Hickey, M., Yu, X., Chau, F., Nonomiya, J. & Lewis, C. (2009). *J Med Chem* **52**, 1255-1258.

Li, L. S., Zhou, Y., Murphy, D. E., Stankovic, N., Zhao, J., Dragovich, P. S., Bertolini, T., Sun, Z., Ayida, B., Tran, C. V., Ruebsam, F., Webber, S. E., Shah, A. M., Tsan, M., Showalter, R. E., Patel, R., Lebrun, L. A., Bartkowski, D. M., Nolan, T. G., Norris, D. A., Kamran, R., Brooks, J., Sergeeva, M. V., Kirkovsky, L., Zhao, Q. & Kissinger, C. R. (2008). *Bioorg Med Chem Lett* **18**, 3446-3455.

Lim, S. P., Koh, J. H., Seh, C. C., Liew, C. W., Davidson, A. D., Chua, L. S., Chandrasekaran, R., Cornvik, T. C., Shi, P. Y. & Lescar, J. (2013). *J Biol Chem* **288**, 31105-31114.

Love, R. A., Parge, H. E., Yu, X., Hickey, M. J., Diehl, W., Gao, J., Wriggers, H., Ekker, A., Wang, L., Thomson, J. A., Dragovich, P. S. & Fuhrman, S. A. (2003). *J Virol* **77**, 7575-7581.

Marcotte, L. L., Wass, A. B., Gohara, D. W., Pathak, H. B., Arnold, J. J., Filman, D. J., Cameron, C. E. & Hogle, J. M. (2007). *J Virol* **81**, 3583-3596.

Martin Hernando, J. I., Ontoria, J. M., Malancona, S., Attenni, B., Fiore, F., Bonelli, F., Koch, U., Di Marco, S., Colarusso, S., Ponzi, S., Gennari, N., Vignetti, S. E., Del Rosario Rico Ferreira, M., Habermann, J., Rowley, M. & Narjes, F. (2009). *ChemMedChem* **4**, 1695-1713.

Maynard, A., Crosby, R. M., Ellis, B., Hamatake, R., Hong, Z., Johns, B. A., Kahler, K. M., Koble, C., Leivers, A., Leivers, M. R., Mathis, A., Peat, A. J., Pouliot, J. J., Roberts, C. D., Samano, V., Schmidt, R. M., Smith, G. K., Spaltenstein, A., Stewart, E. L., Thommes, P., Turner, E. M., Voitenleitner, C., Walker, J. T., Waitt, G., Weatherhead, J., Weaver, K., Williams, S., Wright, L., Xiong, Z. Z., Haigh, D. & Shotwell, J. B. (2014). *J Med Chem* **57**, 1902-1913.

Mosley, R. T., Edwards, T. E., Murakami, E., Lam, A. M., Grice, R. L., Du, J., Sofia, M. J., Furman, P. A. & Otto, M. J. (2012). *J Virol* **86**, 6503-6511.

Moustafa, I. M., Korboukh, V. K., Arnold, J. J., Smidansky, E. D., Marcotte, L. L., Gohara, D. W., Yang, X., Sanchez-Farran, M. A., Filman, D., Maranas, J. K., Boehr, D. D., Hogle, J. M., Colina, C. M. & Cameron, C. E. (2014). *J Biol Chem* **289**, 36229-36248.

Narjes, F., Crescenzi, B., Ferrara, M., Habermann, J., Colarusso, S., Ferreira Mdel, R., Stansfield, I., Mackay, A. C., Conte, I., Ercolani, C., Zaramella, S., Palumbi, M. C., Meuleman, P., Leroux-Roels, G., Giuliano, C., Fiore, F., Di Marco, S., Baiocco, P., Koch, U., Migliaccio, G., Altamura, S., Laufer, R., De Francesco, R. & Rowley, M. (2011). *J Med Chem* **54**, 289-301.

Ng, K. K., Cherney, M. M., Vazquez, A. L., Machin, A., Alonso, J. M., Parra, F. & James, M. N. (2002). *J Biol Chem* **277**, 1381-1387.

Ng, K. K., Pendas-Franco, N., Rojo, J., Boga, J. A., Machin, A., Alonso, J. M. & Parra, F. (2004). *J Biol Chem* **279**, 16638-16645.

Noble, C. G., Lim, S. P., Arora, R., Yokokawa, F., Nilar, S., Seh, C. C., Wright, S. K., Benson, T. E., Smith, P. W. & Shi, P. Y. (2016). *J Biol Chem* **291**, 8541-8548.

Noble, C. G., Lim, S. P., Chen, Y. L., Liew, C. W., Yap, L., Lescar, J. & Shi, P. Y. (2013). *J Virol* **87**, 5291-5295.

O'Farrell, D., Trowbridge, R., Rowlands, D. & Jager, J. (2003). *J Mol Biol* **326**, 1025-1035.

Ontoria, J. M., Rydberg, E. H., Di Marco, S., Tomei, L., Attenni, B., Malancona, S., Martin Hernando, J. I., Gennari, N., Koch, U., Narjes, F., Rowley, M., Summa, V., Carroll, S. S., Olsen, D. B., De Francesco, R., Altamura, S., Migliaccio, G. & Carfi, A. (2009). *J Med Chem* **52**, 5217-5227.

Pan, J., Vakharia, V. N. & Tao, Y. J. (2007). *Proc Natl Acad Sci U S A* **104**, 7385-7390.

Poranen, M. M., Salgado, P. S., Koivunen, M. R., Wright, S., Bamford, D. H., Stuart, D. I. & Grimes, J. M. (2008). *Nucleic Acids Res* **36**, 6633-6644.

Powers, J. P., Piper, D. E., Li, Y., Mayorga, V., Anzola, J., Chen, J. M., Jaen, J. C., Lee, G., Liu, J., Peterson, M. G., Tonn, G. R., Ye, Q., Walker, N. P. & Wang, Z. (2006). *J Med Chem* **49**, 1034-1046.

Qian, X., Hamid, F. M., El Sahili, A., Darwis, D. A., Wong, Y. H., Bhushan, S., Makeyev, E. V. & Lescar, J. (2016). *J Biol Chem* **291**, 9295-9309.

Biology Open • Supplementary information

Ruebsam, F., Murphy, D. E., Tran, C. V., Li, L. S., Zhao, J., Dragovich, P. S., McGuire, H. M., Xiang, A. X., Sun, Z., Ayida, B. K., Blazel, J. K., Kim, S. H., Zhou, Y., Han, Q., Kissinger, C. R., Webber, S. E., Showalter, R. E., Shah, A. M., Tsan, M., Patel, R. A., Thompson, P. A., Lebrun, L. A., Hou, H. J., Kamran, R., Sergeeva, M. V., Bartkowski, D. M., Nolan, T. G., Norris, D. A., Khandurina, J., Brooks, J., Okamoto, E. & Kirkovsky, L. (2009). *Bioorg Med Chem Lett* **19**, 6404-6412.

Ruebsam, F., Webber, S. E., Tran, M. T., Tran, C. V., Murphy, D. E., Zhao, J., Dragovich, P. S., Kim, S. H., Li, L. S., Zhou, Y., Han, Q., Kissinger, C. R., Showalter, R. E., Lardy, M., Shah, A. M., Tsan, M., Patel, R., Lebrun, L. A., Kamran, R., Sergeeva, M. V., Bartkowski, D. M., Nolan, T. G., Norris, D. A. & Kirkovsky, L. (2008). *Bioorg Med Chem Lett* **18**, 3616-3621.

Schoenfeld, R. C., Bourdet, D. L., Brameld, K. A., Chin, E., de Vicente, J., Fung, A., Harris, S. F., Lee, E. K., Le Pogam, S., Leveque, V., Li, J., Lui, A. S., Najera, I., Rajyaguru, S., Sangi, M., Steiner, S., Talamas, F. X., Taygerly, J. P. & Zhao, J. (2013). *J Med Chem* **56**, 8163-8182.

Scrima, N., Caillet-Saguy, C., Ventura, M., Harrus, D., Astier-Gin, T. & Bressanelli, S. (2012). *J Virol* **86**, 7107-7117.

Sholders, A. J. & Peersen, O. B. (2014). *J Mol Biol* **426**, 1407-1419.

Simister, P., Schmitt, M., Geitmann, M., Wicht, O., Danielson, U. H., Klein, R., Bressanelli, S. & Lohmann, V. (2009). *J Virol* **83**, 11926-11939.

Slater, M. J., Amphlett, E. M., Andrews, D. M., Bravi, G., Burton, G., Cheasty, A. G., Corfield, J. A., Ellis, M. R., Fenwick, R. H., Fernandes, S., Guidetti, R., Haigh, D., Hartley, C. D., Howes, P. D., Jackson, D. L., Jarvest, R. L., Lovegrove, V. L., Medhurst, K. J., Parry, N. R., Price, H., Shah, P., Singh, O. M., Stocker, R., Thommes, P., Wilkinson, C. & Wonacott, A. (2007). *J Med Chem* **50**, 897-900.

Surana, P., Satchidanandam, V. & Nair, D. T. (2014). *Nucleic Acids Res* **42**, 2758-2773.

Talamas, F. X., Abbot, S. C., Anand, S., Brameld, K. A., Carter, D. S., Chen, J., Davis, D., de Vicente, J., Fung, A. D., Gong, L., Harris, S. F., Inbar, P., Labadie, S. S., Lee, E. K., Lemoine, R., Le Pogam, S., Leveque, V., Li, J., McIntosh, J., Najera, I., Park, J., Railkar, A., Rajyaguru, S., Sangi, M., Schoenfeld, R. C., Staben, L. R., Tan, Y., Taygerly, J. P., Villasenor, A. G. & Weller, P. E. (2014). *J Med Chem* **57**, 1914-1931.

Talamas, F. X., Ao-Ieong, G., Brameld, K. A., Chin, E., de Vicente, J., Dunn, J. P., Ghate, M., Giannetti, A. M., Harris, S. F., Labadie, S. S., Leveque, V., Li, J., Lui, A. S., McCaleb, K. L., Najera, I., Schoenfeld, R. C., Wang, B. & Wong, A. (2013). *J Med Chem* **56**, 3115-3119.

Tarantino, D., Pezzullo, M., Mastrangelo, E., Croci, R., Rohayem, J., Robel, I., Bolognesi, M. & Milani, M. (2014). *Antiviral Res* **102**, 23-28.

Thompson, A. A., Albertini, R. A. & Peersen, O. B. (2007). *J Mol Biol* **366**, 1459-1474.

Thompson, A. A. & Peersen, O. B. (2004). *EMBO J* **23**, 3462-3471.

van der Linden, L., Vives-Adrian, L., Selisko, B., Ferrer-Orta, C., Liu, X., Lanke, K., Ulferts, R., De Palma, A. M., Tanchis, F., Goris, N., Lefebvre, D., De Clercq, K., Leyssen, P., Lacroix, C., Purstinger, G., Coutard, B., Canard, B., Boehr, D. D., Arnold, J. J., Cameron, C. E., Verdaguer, N., Neyts, J. & van Kuppeveld, F. J. (2015). *PLoS Pathog* **11**, e1004733.

Wang, G., Lei, H., Wang, X., Das, D., Hong, J., Mackinnon, C. H., Coulter, T. S., Montalbetti, C. A., Mears, R., Gai, X., Bailey, S. E., Ruhrmund, D., Hooi, L., Misialek, S., Rajagopalan, P. T., Cheng, R. K., Barker, J. J., Felicetti, B., Schonfeld, D. L., Stoycheva, A., Buckman, B. O., Kossen, K., Seiwert, S. D. & Beigelman, L. (2009). *Bioorg Med Chem Lett* **19**, 4480-4483.

Wang, M., Ng, K. K., Cherney, M. M., Chan, L., Yannopoulos, C. G., Bedard, J., Morin, N., Nguyen-Ba, N., Alaoui-Ismaili, M. H., Bethell, R. C. & James, M. N. (2003). *J Biol Chem* **278**, 9489-9495.

Wright, S., Poranen, M. M., Bamford, D. H., Stuart, D. I. & Grimes, J. M. (2012). *J Virol* **86**, 2837-2849.

Wu, Y., Lou, Z., Miao, Y., Yu, Y., Dong, H., Peng, W., Bartlam, M., Li, X. & Rao, Z. (2010). *Protein Cell* **1**, 491-500.

Yan, S., Appleby, T., Gunic, E., Shim, J. H., Tasu, T., Kim, H., Rong, F., Chen, H., Hamatake, R., Wu, J. Z., Hong, Z. & Yao, N. (2007). *Bioorg Med Chem Lett* **17**, 28-33.

Yan, S., Appleby, T., Larson, G., Wu, J. Z., Hamatake, R., Hong, Z. & Yao, N. (2006). *Bioorg Med Chem Lett* **16**, 5888-5891.

Yan, S., Appleby, T., Larson, G., Wu, J. Z., Hamatake, R. K., Hong, Z. & Yao, N. (2007). *Bioorg Med Chem Lett* **17**, 1991-1995.

Yan, S., Larson, G., Wu, J. Z., Appleby, T., Ding, Y., Hamatake, R., Hong, Z. & Yao, N. (2007). *Bioorg Med Chem Lett* **17**, 63-67.

Yang, H., Hendricks, R. T., Arora, N., Nitzan, D., Yee, C., Lucas, M. C., Yang, Y., Fung, A., Rajyaguru, S., Harris, S. F., Leveque, V. J., Hang, J. Q., Pogam, S. L., Reuter, D. & Tavares, G. A. (2010). *Bioorg Med Chem Lett* **20**, 4614-4619.

Yap, T. L., Xu, T., Chen, Y. L., Malet, H., Egloff, M. P., Canard, B., Vasudevan, S. G. & Lescar, J. (2007). *J Virol* **81**, 4753-4765.

Zhao, Y., Soh, T. S., Zheng, J., Chan, K. W., Phoo, W. W., Lee, C. C., Tay, M. Y., Swaminathan, K., Cornvik, T. C., Lim, S. P., Shi, P. Y., Lescar, J., Vasudevan, S. G. & Luo, D. (2015). *PLoS Pathog* **11**, e1004682.

Zhou, Y., Webber, S. E., Murphy, D. E., Li, L. S., Dragovich, P. S., Tran, C. V., Sun, Z., Ruebsam, F., Shah, A. M., Tsan, M., Showalter, R. E., Patel, R., Li, B., Zhao, Q., Han, Q., Hermann, T., Kissinger, C. R., Lebrun, L., Sergeeva, M. V. & Kirkovsky, L. (2008). *Bioorg Med Chem Lett* **18**, 1413-1418.
